# Effects of *KEAP1* Silencing on NRF2 and NOTCH Pathways in SCLC Cell Lines

**DOI:** 10.3390/cancers16101885

**Published:** 2024-05-15

**Authors:** Federico Pio Fabrizio, Angelo Sparaneo, Giusy Gorgoglione, Pierpaolo Battista, Flavia Centra, Francesco Delli Muti, Domenico Trombetta, Antonella Centonza, Paolo Graziano, Antonio Rossi, Vito Michele Fazio, Lucia Anna Muscarella

**Affiliations:** 1 Laboratory of Oncology, Fondazione IRCCS Casa Sollievo della Sofferenza, 71013 San Giovanni Rotondo, Italy; a.sparaneo@operapadrepio.it (A.S.); gorgoglione.giusy@gmail.com (G.G.); pierpaolonunzio.battista@outlook.com (P.B.); f.centra@operapadrepio.it (F.C.); f.dellimuti@operapadrepio.it (F.D.M.); d.trombetta@operapadrepio.it (D.T.); vitomichele.fazio@ift.cnr.it (V.M.F.); 2 Department of Experimental Oncology, IEO European Institute of Oncology IRCCS, 20139 Milan, Italy; 3 Department of Oncology and Hemato-Oncology, University of Milan, 20122 Milan, Italy; 4 Oncology Unit, Fondazione IRCCS Casa Sollievo della Sofferenza, 71013 San Giovanni Rotondo, Italy; antocento86@gmail.com; 5 Pathology Unit, Fondazione IRCCS Casa Sollievo della Sofferenza, 71013 San Giovanni Rotondo, Italy; paologratz@gmail.com; 6 Oncology Center of Excellence, Therapeutic Science & Strategy Unit, IQVIA, 20124 Milan, Italy; 7 Department of Medicine, Laboratory of Molecular Medicine and Biotechnology, University Campus Bio-Medico of Rome, 00128 Rome, Italy; 8 Institute of Translational Pharmacology, National Research Council of Italy (CNR), 00185 Rome, Italy

**Keywords:** SCLC, KEAP1, NRF2, NOTCH, HES1, DLL-3

## Abstract

**Simple Summary:**

The role of neurogenic locus notch homolog protein 1 (NOTCH1)- and nuclear factor erythroid 2-related factor 2 (NRF2)-related pathways is highly heterogeneous in small cell lung cancer (SCLC) and, when they are both abnormally activated, they can synergistically cause neoplastic proliferation. In this paper, we investigate the possible role of *KEAP1* silencing in NRF2 and NOTCH axis deregulation in SCLC by evaluating its impact on the modulation of cellular defense systems, cell growth, and differentiation. We also investigate the impact of these systems’ impairment in response to conventional chemotherapies and NOTCH inhibitors in silenced SCLC cell lines with different Kelch-like ECH-associated protein 1 (*KEAP1*) alterations.

**Abstract:**

The KEAP1/NRF2 pathway is a master regulator of several redox-sensitive genes implicated in the resistance of tumor cells against therapeutic drugs. The dysfunction of the KEAP1/NRF2 system has been correlated with neoplastic patients’ outcomes and responses to conventional therapies. In lung tumors, the growth and the progression of cancer cells may also involve the intersection between the molecular NRF2/KEAP1 axis and other pathways, including NOTCH, with implications for antioxidant protection, survival of cancer cells, and drug resistance to therapies. At present, the data concerning the mechanism of aberrant NRF2/NOTCH crosstalk as well as its genetic and epigenetic basis in SCLC are incomplete. To better clarify this point and elucidate the contribution of NRF2/NOTCH crosstalk deregulation in tumorigenesis of SCLC, we investigated genetic and epigenetic dysfunctions of the *KEAP1* gene in a subset of SCLC cell lines. Moreover, we assessed its impact on SCLC cells’ response to conventional chemotherapies (etoposide, cisplatin, and their combination) and NOTCH inhibitor treatments using DAPT, a γ-secretase inhibitor (GSI). We demonstrated that the KEAP1/NRF2 axis is epigenetically controlled in SCLC cell lines and that silencing of *KEAP1* by siRNA induced the upregulation of NRF2 with a consequent increase in SCLC cells’ chemoresistance under cisplatin and etoposide treatment. Moreover, *KEAP1* modulation also interfered with NOTCH1, HES1, and DLL3 transcription. Our preliminary data provide new insights about the downstream effects of KEAP1 dysfunction on NRF2 and NOTCH deregulation in this type of tumor and corroborate the hypothesis of a cooperation of these two pathways in the tumorigenesis of SCLC.

## 1. Introduction

SCLC is a high-grade neuroendocrine carcinoma that includes approximately 15% of all lung cancer that is diagnosed in the world annually. In about 70% of these cases, the diagnosis is performed at the extensive stage (ES) of disease. The estimation of the overall 5-year survival rate for patients with SCLC is about 6%, which is halved after 10 years [1,2]. The etiology of SCLC is heavily related to smoking habit, grouping almost all patients that have a smoking history, and appears to be characterized by rapid growth and multiple organ metastasis [3,4]. The initial strong response of SCLC to conventional chemotherapy (approximately 60–70% response rates) and radiation is counteracted by its resistance to second-line and subsequent therapies developed after cancer recurrence [5].

Most recently, a large number of studies have shown promising antitumor activity from first-line immunotherapy drugs plus platinum-based chemotherapy. This has resulted in a significant improvement in SCLC patients with extensive disease in terms of disease-free survival (PFS) and overall survival (OS) versus chemotherapy alone [6,7,8].

Given its aggressive behavior that drives tumor resistance and justifies its poor prognosis, SCLC is characterized by genomic instability and a high mutational burden, increasing a very complex molecular profile that suffers from poor availability of biological samples to use in cancer studies [9]. The loss of function of retinoblastoma 1 (*RB1*) and tumor protein p53 are considered the most frequent alterations found in SCLC [10,11]. Less recurrent genetic events such as gain of function and copy-number amplifications have also been reported in the myelocytomatosis oncogene (*MYC)*, phosphoinositide 3-kinase (*PI3K*) pathway genes, phosphatase and tensin homolog (*PTEN*), and phosphatidylinositol-4,5-bisphosphate 3-kinase, catalytic subunit alpha (*PIK3CA*). Rat sarcoma (*RAS*)/mitogen-activated protein kinase (*MAPK*) upregulation also concurred in post-translational modifications, as well as histone–lysine N-methyltransferase 2D, cyclic AMP response element binding protein, and *NOTCH1* mutations. The latter tends to be clustered in the extracellular domain of NOTCH1 protein with an incidence ranging from 5% to 15%, thus supporting the role of NOTCH1 in tumor growth and acquisition of neuroendocrine features [2,10,11,12]. The *NOTCH* genes encode a family of highly conserved cell surface receptors that drives a complex signaling pathway including a large number of ligands, negative and positive modifiers, and transcription factors [13,14]. There are four functional NOTCH ligands in mammals, all of which are also single-pass transmembrane proteins: DLL1 and DLL4, which are part of the delta family of ligands, and jagged canonical notch ligand 1 (Jag1) and Jag2, which are members of the serrate family of ligands. The delta-like ligand 3 (DLL3) *DLL3* gene appears to encode a decoy ligand, as phenotypes observed in DLL3-deficient mice were consistent with *NOTCH* gain of function [15]. The DLL3 ligand has been found to be highly expressed on the surface of SCLC cells and other high-grade neuroendocrine tumors and is becoming one of the most interesting targets of experimental therapies in SCLC with antibody–drug conjugates (ADCs), T-cell engager (TCE) molecules, and chimeric antigen receptor (CAR) [16]. The NOTCH-driven cell-signaling pathway is involved in antioxidant protection, the survival of cancer cells, and resistance to drugs, as well as chemotherapy and immunotherapy, and contributes to intratumoral heterogeneity [13,17].

In the past decade, the efficacy of targeting key “growth drivers” in cancer treatment of small subsets of lung cancers has improved, encouraging the investigation of novel target proteins and cellular mechanisms that are selectively expressed and/or that undergo genomic alterations in SCLC cancer cells.

In these contexts, the growth and the progression of SCLC was demonstrated to be related to the well-known molecular KEAP1/NRF2 axis, a master regulator of antioxidants and cellular stress responses, also implicated in the resistance of tumor cells against chemo- and radiotherapies [18,19]. The NRF2 is a key regulator of the cell’s adaptative response to radical oxidant species and xenobiotics through the interaction with its negative regulator, Keap1, and contributes to cancer development, progression, and chemoresistance [18,20,21]. Molecular dysfunction of the KEAP1/NRF2 axis was mainly investigated in NSCLC, whereas few papers have elucidated the impact of this mechanism deregulation in SCLCs [18].

Both NOTCH and NRF2 are transcription factors, and their related pathways were discovered and described independently. However, recent emerging data have shown that the NRF2–NOTCH crosstalk influences cytoprotection and enhances the maintenance of cellular homeostasis and tissue organization, through actions on cell proliferation kinetics and cell fate determinants of stem cell renewal and development of differentiated cell types. Detailed mechanisms for the downregulation of this crosstalk remain to be characterized [22,23]. Interestingly, NRF2 and NOTCH signaling were also reported to mutually regulate each other, since the *NFE2L2* gene (codifying the NRF2 protein) is a downstream gene modulated by NOTCH signaling and the *NOTCH1* gene is a target of NRF2 [24]. Moreover, in the basal cell state, NRF2 is modulated by the KEAP1 protein, whose interaction induces its ubiquitination through which NRF2 is degraded by the Cul3-Rbx1-E3 ligase ubiquitin–proteasome complex. In the activated state, de novo synthesized NRF2 protein accumulates in the nucleus, where it promotes the transcription of several *ARE* genes, including *NOTCH1* [22,23,25,26].

These observations led to the speculative notion that the impairment of aberrant molecular KEAP1/NRF2 and NOTCH crosstalk by genetic and epigenetic factors in these genes might impact the complex landscape of SCLC and exert a critical role in oxidative stress, metabolism, and cell fate determination of neuroendocrine lung tumors and SCLC. More specifically, if an aberrant crosstalk between NRF2 and NOTCH exists in SCLC, it should be modulated by genetic lesions and aberrant DNA methylation in key genes of their pathways, such as *KEAP1*, which should establish a synergic and critical effect on tumorigenesis and progression of the disease [22,23,27].

In this paper, we aim to better understand the features of the *KEAP1* genetic alterations and aberrant DNA methylation in SCLC cell lines and their effects on the modulation of NRF2 and NOTCH pathways, thus clarifying their significance in SCLC tumorigenesis. Moreover, we evaluate the response of SCLC cells to conventional therapies under *KEAP1* silencing, such as chemotherapies with cisplatin and etoposide alone and in combination, as well as to NOTCH inhibition using (N-[N-(3,5-difluorophenacetyl)-L-alanyl]-S-phenylglycine t-butyl ester DAPT molecules.

## 2. Material and Methods

### 2.1. DNA and RNA from SCLC Cell Lines

Nucleic acids were from Hcc33, H1963, N417, H1184, H209, and H69V SCLC cell lines (American Type Culture Collection, Manassas, VA, USA), whereas GLC1 and GLC2 were from UMCG, Department of Genetics, Groningen, the Netherlands (Dr. Klaas Kok, PhD). DNA was extracted from each cell line using the standard procedure with phenol–chloroform. RNA was extracted using Trizol reagent (ThermoFisher Scientific, Waltham, MA, USA), according to the manufacturer’s instructions. Both DNA and RNA concentrations were estimated using a Qubit™ 3.0 Fluorimeter (ThermoFisher Scientific, Waltham, MA, USA).

### 2.2. Cell Cultures

N417, H1184, H209, and H69V SCLC (American Type Culture Collection, Manassas, VA, USA) were cultured in RPMI 1640 medium supplemented with 10% fetal bovine serum (FBS), 100 U/mL penicillin, and 100 U/mL streptomycin, and maintained at 37 °C in a 5% CO_2_ incubator. All the cell culture products were purchased from Euroclone (Milan, Italy).

### 2.3. Point Mutation Detection and CNV Analysis

Exon/intron gene structures were obtained from NCBI/Genbank databases and the set of primers used for genetic screening was designed to cover the entire region of the double glycine repeat (DGR) domain of the *KEAP1* gene (NM_203500), the exon 2 of the *NFE2LE* gene (NM_006164), and the *NOTCH1* coding region (NM_017617). PCR amplification of each fragment was performed using a Gene Amp PCR System 9700 thermal cycler (Applied Biosystem, Foster City, CA, USA) and sequenced using the Big Dye Terminator Ready Reaction mix v. 1.1 on an ABI 3100 sequence detection system with Sequencing Analysis software v.3.7 (Applied Biosystems, Foster City, CA, USA). *KEAP1* copy number variation (CNV) for SCLC cell lines using genomic DNA from tumor cell lines for four microsatellite markers flanking the *KEAP1* gene (D19S865, DM1, D19S906, D19S840) was assessed as previously reported and combined with data from the SNP array from http://cancer.sanger.ac.uk/cancergenome/projects/cosmic/ and http://www.ncbi.nlm.nih.gov/geo/ (accessed on 23 April 2024) [28].

### 2.4. DNA Sodium Bisulfite Conversion and Quantitative Methylation Analysis (QMSP)

Bisulfite conversion of DNA and QMSP investigations required one microgram of DNA extracted from cell lines, subjected to bisulfite treatment and DNA purification using the Epitect Bisulfite kit (QIAGEN, Hilden, Germany), according to the manufacturer’s instructions. Bisulfite-modified DNA was then used as a template for QMSP to detect converted DNA. Calibration curves for both target and reference genes were constructed using serial dilutions (0.009–90 ng) of commercially available fully methylated DNA (CpGenome Universal Methylated DNA, Millipore, Chemicon, cat#S7821, Bedford, MA, USA). Amplification reactions were carried out in triplicate in 384-well plates and in a volume of 10 μL that contained 50 ng of bisulfite-modified DNA on an ABI PRISM 7900 Sequence Detection System and were analyzed using SDS 2.1.1 software (Applied Biosystems, Foster City, CA, USA). PCR primers were used to amplify DNA target regions and were designed in silico through MethPrimer for both actin beta (*ACTB*) and *KEAP1*. Primer/probe sets for the *KEAP1* promoter region and for the unmethylated promoter region of the *ACTB* reference gene were as previously described and are reported in Supplementary Table S1. Methylation levels were finally calculated as the ratio of *KEAP1* to *ACTB* and then multiplied by 1000 for easier tabulation (average value of triplicates of *KEAP1*/average value of triplicates of *ACTB* × 1000) [28,29].

### 2.5. Gene Expression Quantification by RT-qPCR

PCR fragments of KEAP1, NFE2L2, AKR1C1, NQO1, TXNRD1 NOTCH1, hairy and enhancer of split 1 (HES1), DLL3 transcripts, and the housekeeping RPLPO transcript were amplified using the TaqMan assays listed in Supplementary Table S2 and were cloned in StrataCloneTM PCR Cloning Vector pSC-A (Stratagene, Milan, Italy). Mini-prep cultures were grown in 5 mL of LB–ampicillin broth. Plasmid DNA from the selected transformed cells was isolated using the QIAprep^®^ Spin Miniprep Kit (Qiagen). Five plasmid dilutions in the range of 1 × 10^6^ copies to 1 × 10^2^ copies were used to construct the standard curves for real-time PCR. First-strand cDNA synthesis from 1 μg of total RNA extracted from SCLC cell lines was carried out with SuperScript III First-Strand Synthesis (Thermo Fisher, Invitrogen Division, Carlsbad, CA, USA) using a gene expression amplification mixture containing 2.5× TaqMan^®^ Universal PCR Master Mix (Thermo Fisher Scientific, Waltham, MA, USA), 250 nM of TaqMan™ Gene Expression Assay with TaqMan probe, and 1 μL of template cDNA or plasmid product (serial dilutions). Reactions were run on an ABI PRISM 7900HT Sequence Detection System (Thermo Fisher Scientific, Waltham, MA, USA). Protocol conditions were as follows: 10′ at 95 °C, 40 cycles at 95 °C for 15″ and 60 °C for 60″. Calibration curves were constructed and sample concentration was calculated using the plasmid standard curve, resulting in plasmid concentrations expressed as a copy number of corresponding standard molecules. The relative sample amount was expressed as a ratio marker ([Target/Housekeeping] × 1000 for easier tabulation).

### 2.6. Protein Extraction and Western Blotting

Cells cultures were solubilized in at least ten volumes of RIPA buffer (25 mM Tris–HCl, pH 7.6; 150 mM NaCl; 1% Triton X-100; 1% sodium deoxycholate; 0.1% SDS) with a cocktail of protease inhibitors (https://lifescience.roche.com, accessed on 23 April 2024). The lysis was performed on ice for 60′ and the samples were then centrifuged at 22,000× *g* for 45′. The protein content of the supernatant was measured with a bicinchoninic acid (BCA) Protein Assay Kit (https://www.thermofisher.com/order/catalog/product/23225, accessed on 23 April 2024). Equal amounts of protein samples were separated by 12% Tris–glycine–SDS–PAGE and transferred to polyvinylidene fluoride (PVDF) membranes (http://www.merckmillipore.com, accessed on 23 April 2024). Membranes were saturated with 5% fat-free milk and were incubated with primary antibodies overnight at 4 °C, washed, and incubated with peroxidase-conjugated secondary antibodies for 60′ at room temperature. Reactive proteins were revealed with an enhanced chemiluminescent detection system (ECL Plus, ThermoScientific, Rockford, IL, USA) and visualized on a Chemidoc XRS imaging system (Biorad, Hercules, CA, USA).

### 2.7. KEAP1 Silencing

*KEAP1* short interfering RNA (siRNA) duplexes specific for human *KEAP1* (NM_012289.3) were purchased from Thermo Scientific (Carlsbad, CA, USA, Silencer Select, s18981, *KEAP1* exon4). A scrambled siRNA purchased from Thermo Scientific (Carlsbad, CA, USA) was used as a negative control (CTRL siRNA, Silencer Select). RNA interference (RNAi) experiments were performed with transient transfection for 48 h using the RNAiMAX Lipofectamine (Invitrogen, Milan, Italy) transfection protocol [29,30].

### 2.8. Immunofluorescence and Confocal Microscopy

Cell cultures were plated on coverslips and fixed in 4% paraformaldehyde, washed in phosphate-buffered saline (PBS), and permeabilized with 0.3% Triton X-100 in PBS. After blocking with 1% BSA in PBS, cells were incubated with primary antibodies for 120′ at RT. After washing in PBS, cells were incubated for 60′ at RT with Alexa-conjugated secondary antibodies and Phalloidin 647 (Alexa Fluor, Thermo Scientific) to stain the cytoskeleton. Coverslips were mounted on slides using a mounting medium (PBS, 50% glycerol, 0.1% N-propyl-gallate) and examined using a confocal microscope (TCS SP8, Leica). Nuclei were stained with 4′,6-diamidino-2-phenylindole (DAPI). Once captured, the auto-contrast function was applied to all the images using Adobe Photoshop CS5 to create a more accurate tonal and color-corrected workflow.

### 2.9. Antibodies

The following primary antibodies were used for the in vitro studies: anti-KEAP1 polyclonal (1:800, Proteintech, Chicago, IL, USA), anti-AKR1C1 monoclonal (1:1000, Proteintech, Chicago, IL, USA), anti-TXNRD1 polyclonal (1:1000, Proteintech, Chicago, IL, USA), anti-NRF2 polyclonal (1:500, Proteintech, Chicago, IL, USA), anti-NOTCH1 monoclonal (1:1000, CST, Danvers, MA, USA), anti-epithelial cadherin (E-Cadherin, 1:1000, CST, Danvers, MA, USA), anti-HES1 (1:1000, CST, Danvers, MA, USA), anti-caspase 3 (CAS-3) full length (1:1000, CST, Danvers, MA, USA), anti-CAS-3 cleaved (1:1000, CST, Danvers, MA, USA), anti-B-cell lymphoma 2 (BCL2, 1:500, DAKO, Trappes, France), anti c-Myc (1:1000, CST, Danvers, MA, USA), anti-β-actin (1:10,000, Sigma-Aldrich, St. Louis, MO, USA). The following secondary antibodies were used for the in vitro studies: horseradish peroxidase (HRP)-conjugated donkey anti-goat and donkey anti-mouse IgG (https://www.sigmaaldrich.com/IT/it/product/mm/ap192p, accessed on 23 April 2024) for Western blotting and Alexa-conjugated secondary antibodies 488 and 594 (Alexa Fluor, Invitrogen, Thermo Fisher Scientific, Waltham, MA, USA) for immunofluorescence.

### 2.10. Pharmacological Treatments

The best concentration of chemotherapies and γ-secretase inhibitor (IC50) was determined for the *KEAP1* silencing experiments under drug treatments (Figure S1). The H69V and H1184 cell lines were treated with cisplatin (Sigma Aldrich, St. Louis, MO, USA), etoposide (Sigma Aldrich, St. Louis, MO, USA), or both, and DAPT (Selleck Chemicals LLC, Houston, TX, USA) via adding drugs dissolved in PBS to the medium at the concentrations indicated. Etoposide works by blocking topoisomerase 2, which is necessary for cancer cells’ division. If this enzyme is blocked, the cell’s DNA is tangled up and the cell cannot divide. Cisplatin works by interfering with DNA replication and killing the fastest proliferating cells.

For DAPT treatment, *KEAP1* silenced cells were treated with increasing concentrations of DAPT alone (50 μM, 65 μM 85 μM, 100 μM, 24 h) that inhibited γ-secretase inhibitor (GSI) production and enhanced the apoptotic effects by blocking the NOTCH signaling pathway. According to Cao and colleagues [31], it was established that the best concentration of γ-secretase inhibitor to use in *KEAP1* silencing experiments is 100 μM (Figure S2).

### 2.11. Cell Viability Assay

The viability and proliferation of cell lines were analyzed using PrestoBlue™ Cell Viability Reagent (Thermo Fisher Scientific, Waltham, MA, USA). Cell lines were cultured into a 96 multi-well plate and, the next day, PrestoBlue was added directly to the culture medium (10% *v*/*v*) of each sample. After 120′ of incubation at 37 °C, fluorescence measurement (560 nm/590 nm) was performed using a BioTek Microplate Reader (Cytation 3, BioTek Instruments, Inc., Winooski, VT, USA).

### 2.12. Statistical Analysis

Data are expressed as mean ± SE of the number (*n*) of independent experiments performed on different cell and nucleic acid preparations. For each experiment, at least three different wells or mRNA expression points were analyzed. Statistically significant differences were computed using Student’s *t*-test, the significance level being set at *p* < 0.05. All statistical analyses were performed using GraphPad Prism statistical software (GraphPad Prism 5, Boston, MA, USA).

## 3. Results

### 3.1. Functional Effects of KEAP1 Genetic Alterations and Aberrant DNA Methylation on NRF2 Axis in SCLC Cell Lines

**Figure 1:**
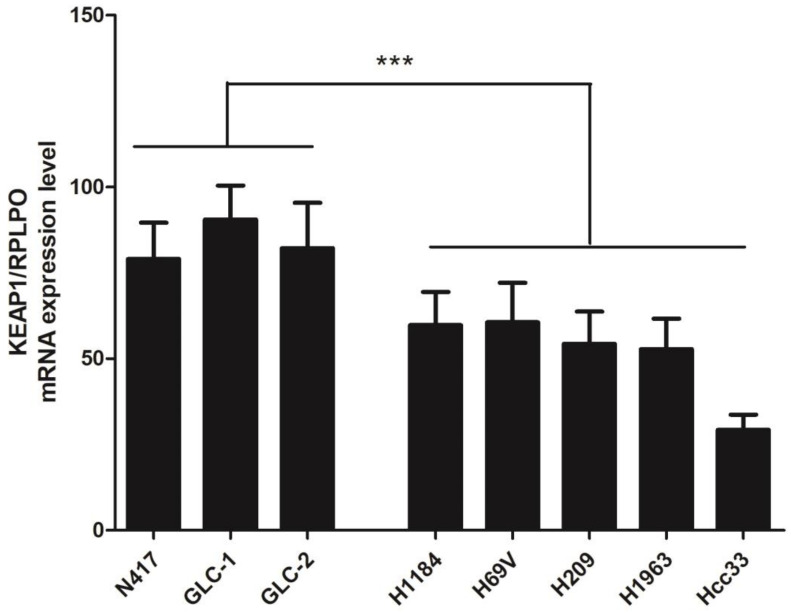
Expression level analysis (±standard error mean) of the *KEAP1* gene transcript determined via RT-qPCR. The relative quantification is expressed as ratio marker (*KEAP1*/*RPLPO*). *** *p* < 0.001 (Student’s *t*-test).

A total of seven SCLC cell lines were selected after performing a comprehensive *KEAP1* and *NFE2L2* molecular profile, and a first explorative analysis of KEAP1 transcript level was assessed via RT-qPCR [19]. The KEAP1 mRNA was significantly downregulated in the four cancer cell lines H1184, H69V, H209, and H1963, having a mutation (H1184, p.Gly364Cys) or a hypermethylation in the *KEAP1* promoter gene (H69V, H209, H1963) or an LOH at the *KEAP1* locus (Hcc33) in comparison with the N417, GLC1, and GLC2 cell lines (*p* = 0.0007, *t*-test), showing no genetic or DNA methylation alterations in *KEAP1* or *NFE2L2* genes (Figure 1).

**Figure 2:**
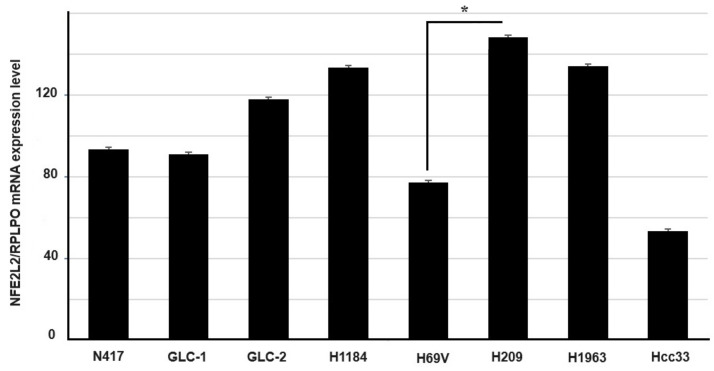
Expression level analysis (±standard error mean) of the *NFE2L2* gene determined via RT-qPCR. The relative quantification is expressed as ratio marker (*NFE2L2*/*RPLPO*). * *p* < 0.05 (Student’s *t*-test).

The mRNA expression levels of NRF2 were evaluated in the same set of SCLC cells. Expression in H209 (CNV = 4) appeared to be higher than those levels observed in the other cell lines and there was a statistically significant difference when compared with the haploid H69V cell line (CNV = 1, *p* = 0.005 *t*-test) (Figure 2).

**Figure 3:**
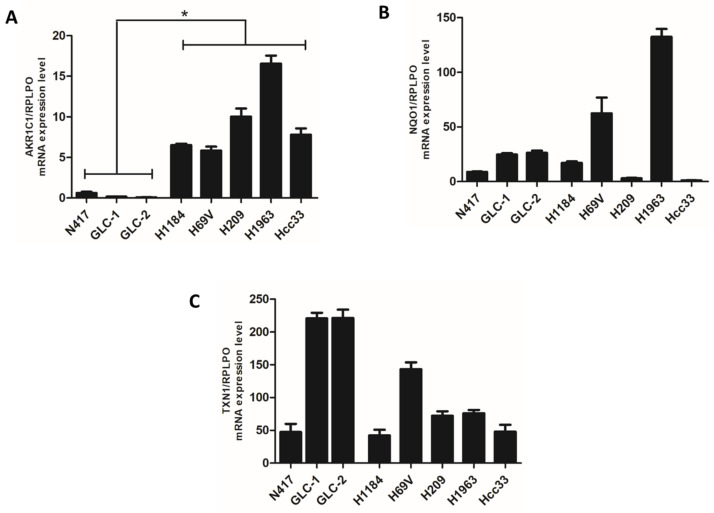
Comparison of mRNA expression level (±standard error mean) of NRF2 target enzymes (**A**) AKR1C1, (**B**) NQO1, and (**C**) TXN1 between SCLC cells having genetic/DNA methylation alterations of the *KEAP1* gene and N417 wild-type SCLC lines. * *p* < 0.05 (Student’s *t*-test).

Finally, we investigated the mRNA expression levels of AKR1C1, TXN1, and NQO1, three different ARE genes involved in the NRF2 cell mechanism defense system, whose expression level was directly modulated by NRF2 activity. A significant abundance of transcripts of the *AKR1C1* gene in *KEAP1* genetic/DNA methylation deregulated cell lines was observed (Figure 3).

**Figure 4:**
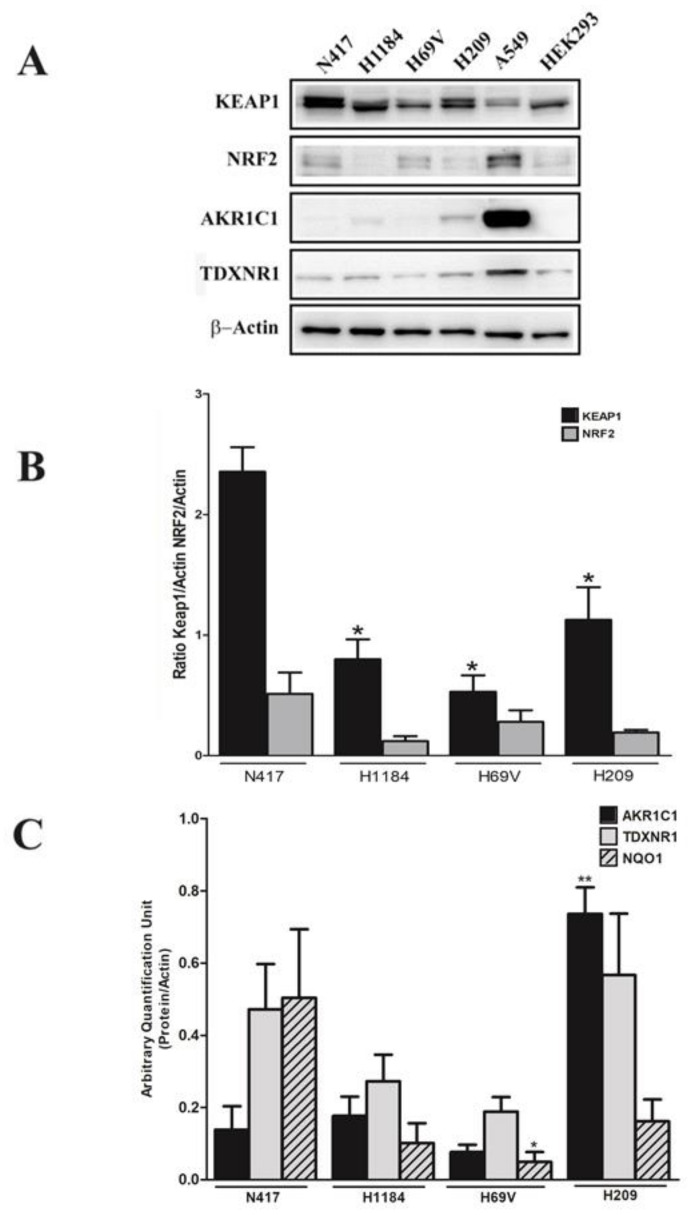
(**A**) Representative immunoblots showing a comparison between the expression levels of the KEAP1, NRF2, AKR1C1, and TXNRD1 proteins in N417, H1184, H69V, and H209 cell lines. Original western blots are shown in the Supplementary Materials as File S1. (**B**) Histograms showing the expression levels of the KEAP1 and NRF2 proteins normalized to actin. (**C**) Histograms showing the expression levels of the AKR1C1, TDXNR1, and NQO1 proteins normalized to actin (*n* = 4). * *p* < 0.05, ** *p* < 0.01, (Student’s *t*-test using N417 wild-type KEAP1 control). The A549 and HEK293 cells were used as a positive control for NRF2 and KEAP1 expression.

The KEAP1/NRF2 axis status was also investigated in SCLC cell lines through evaluating the protein levels of KEAP1, NRF2, and NRF2 targets via performing Western blot analysis on the available cell lines N417, H1184, H69V, and H209. A significant lower KEAP1 protein level was observed in cell lines that had genetic/DNA methylation deregulation of *KEAP1* (Figure 4A,B) compared with the N417 wild-type cell line, whereas variable levels of NRF2 protein and its targets were obtained. High expression of AKR1C1 in H209 and NQO1 in H69V was observed (Figure 4C).

### 3.2. Cellular Localization of KEAP1 and NRF2 in SCLC Cell Lines

**Figure 5:**
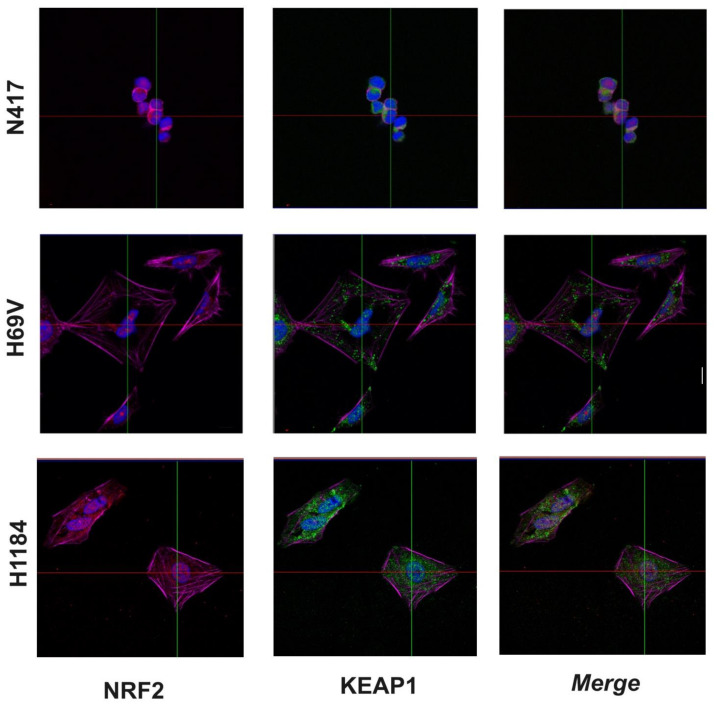
Subcellular localizations of KEAP1 and NRF2 proteins in N417, H69V, and H1184 SCLC cell lines evaluated using immunofluorescence. The proteins’ subcellular localizations were examined via immunostaining with anti-NRF2 (red), anti-KEAP1 (green), anti-actin antibodies, and DAPI and examined via confocal microscopic. Blue = nucleus. Magenta = actin. Scale bar: 20 µm.

The cellular localization patterns of the KEAP1 and NRF2 proteins were investigated in N417 (with no alterations in *KEAP1* and *NFE2L2*), H69V (*KEAP1*-methylated), and H1184 (with the *KEAP1* p.Gly364Cys mutation) SCLC cell lines via immunofluorescence analysis. In the N417 cell line, KEAP1 (green signal) and NRF2 (red signal) were mostly expressed in the cytosol, while in the H69V cell line, a predominantly nuclear localization of NRF2 was observed, thus suggesting an enhancement of its transcriptional activity on the antioxidant response element (ARE)-genes. The same pattern of KEAP1 and NRF2 cellular localization was observed in the H1184 cell line (Figure 5).

### 3.3. Effect of KEAP1 Silencing on KEAP1/NRF2 Pathway in SCLC Cell Lines

*KEAP1* silencing by siRNA was assessed to evaluate the effects of KEAP1 impedance on protein level modulation of KEAP1, NRF2, and its dependent target genes *AKR1C1* and *TDXN1*. Transcripts and protein levels were measured in N417, H1184, and H69V SCLC cell lines via Western blotting.

**Figure 6:**
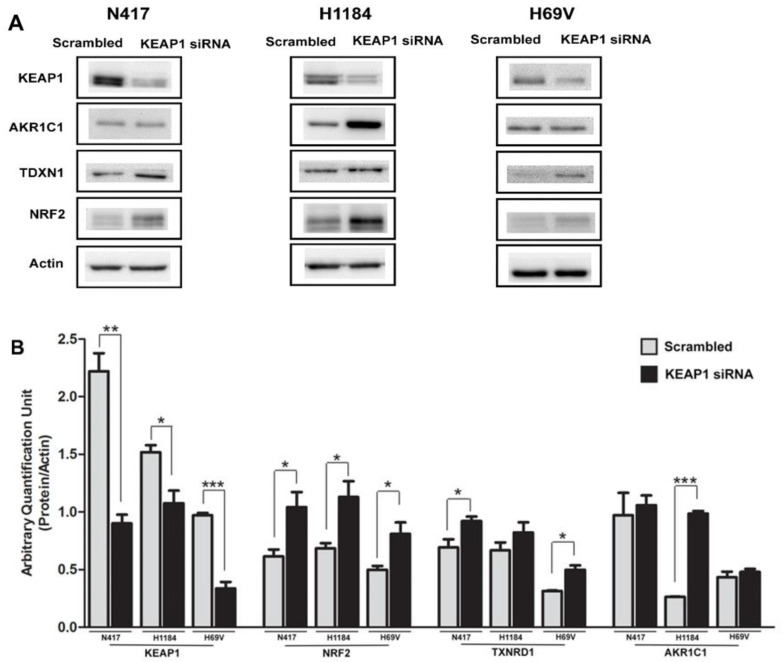
(**A**) Representative immunoblots showing expression levels of KEAP1, NRF2, AKR1C1, and TXNRD1 in N417, H1184, and H69V cell lines after KEAP1 inhibition by specific *KEAP1* siRNA. Scrambled siRNA was employed as the control. Original western blots are shown in the Supplementary Materials as File S1. (**B**) Histograms showing the expression levels of the proteins normalized to actin (N = 4). * *p* < 0.05, ** *p* < 0.001, *** *p* < 0.0001, *t*-test.

In all cell lines, it was clearly observed that a variation of KEAP1 protein level produced a significant increase of NRF2 and its target proteins levels (*p* < 0.05, *t*-test). By contrast, the variation in AKR1C1 was evident in the H1184 cell line, whereas the increase in TXNRD1 protein expression was observed only in N417 and H69V cells (Figure 6).

Similarly to the *KEAP1*-silencing effect of SiRNA, the effects of aberrant promoter DNA methylation of the *KEAP1* gene on its transcript levels were previously reported in the H69V SCLC cell line using 5-AZA-CdR (*p* < 0.05, *t*-test); a progressive increase in KEAP1 transcript abundance was observed after 48 h (*p* < 0.05) and 72 h (*p* < 0.01) and was shown to correlate with decreased *KEAP1* promoter methylation at 48 h (*p* < 0.001) and at 72 h (*p* < 0.01) [32].

### 3.4. Effects of KEAP1 Silencing on NOTCH Pathway

**Figure 7:**
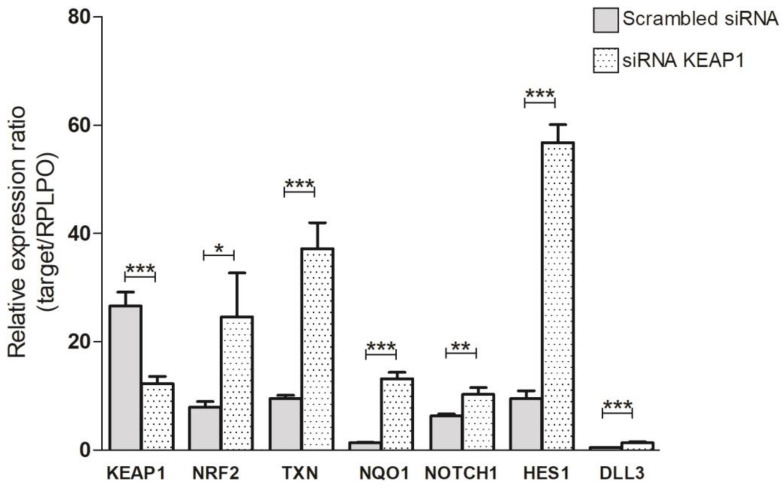
Variation in transcript levels of *KEAP1, NRF2, TXN1, NQO1, NOTCH1*, *HES1*, and *DLL3* in H69V cell line under *KEAP1* silencing * *p* < 0.05,** *p* < 0.01,*** *p* < 0.001 (Student’s *t*-test).

To investigate the role of KEAP1 in NOTCH pathway modulation in SCLC, we first evaluated the NOTCH1 protein levels in the collection of available SCLC cell lines. A detectable NOTCH1 protein level was found only in H69V and H1184 cell lines, thus suggesting these specific cell lines as most suitable for *KEAP1*-silencing experiments (Figure S3). Fluctuations in transcript levels and protein levels were monitored in both *KEAP1*-silenced H69V and H1184 cell lines via RT-qPCR and Western blotting, respectively (Figure 7).

**Figure 8:**
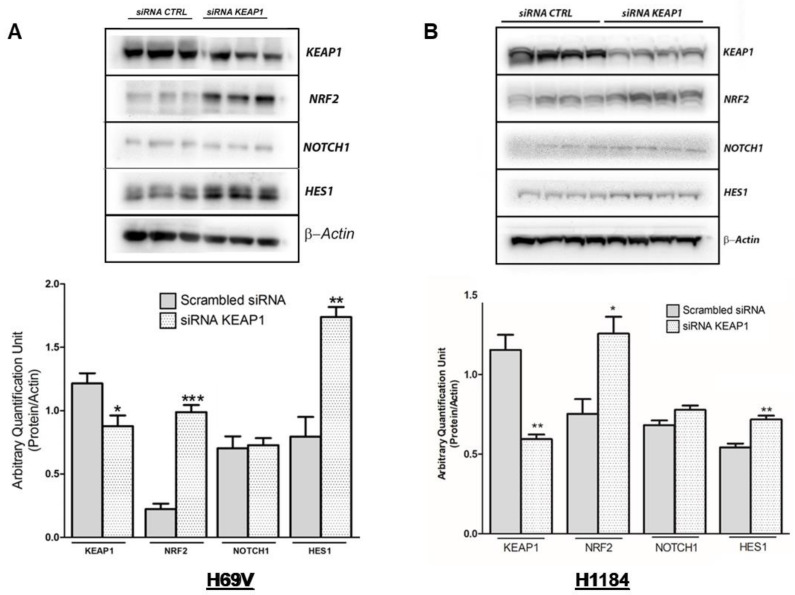
Representative immunoblots and protein levels of KEAP1, NOTCH1, NRF2, and HES1 in (**A**) H69V and (**B**) H1184 cell lines under *KEAP1* silencing, normalized to actin. * *p* < 0.05, ** *p* < 0.01, *** *p* < 0.001 (Student’s *t*-test). Original western blots are shown in the Supplementary Materials as File S1.

In H69V cell lines, it was clearly observed that *KEAP1* silencing not only impacted NRF2 and its target TXN and NQO1 transcript levels but also induced a significant increase in mRNA levels of NOTCH1 and its targets HES1 and DLL3 (Figure 7). Only HES1 resulted in significantly increased protein levels in H69V and H1184 cell lines (Figure 8).

### 3.5. Effect of KEAP1 Silencing in SCLC Cell Lines under Etoposide and Cisplatin Treatment

**Figure 9:**
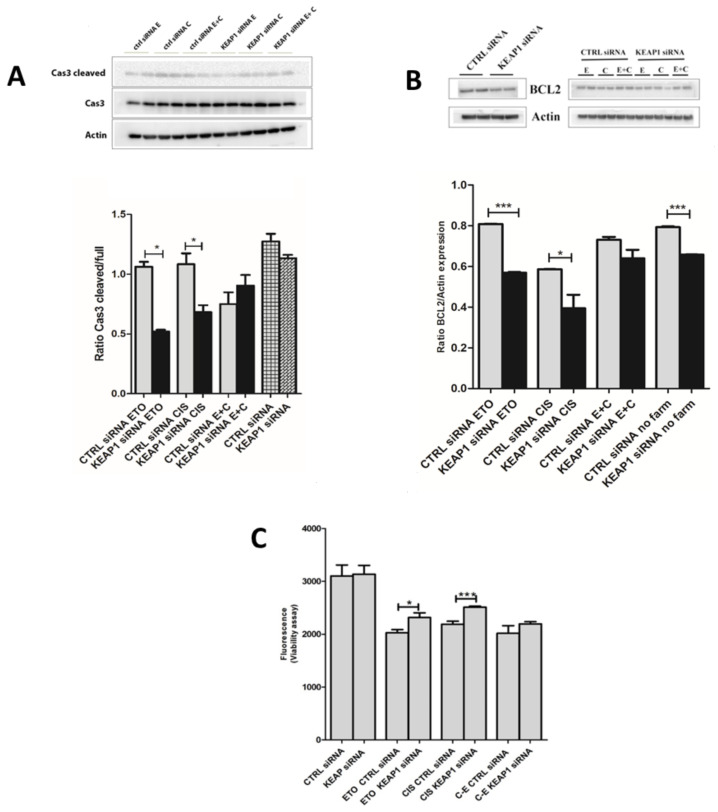
Representative immunoblots and protein levels of (**A**) caspase 3 and (**B**) BCL2 expression in H69V *KEAP1*-silenced cell line under pharmacological treatment with etoposide and cisplatin. Original western blots are shown in the Supplementary Materials as File S1. (**C**) The viability of H69V cell line after *KEAP1* silencing was tested under cisplatin and etoposide treatment. * *p* < 0.05, *** *p* < 0.001 (Student’s *t*-test). E and ETO, etoposide; C and CIS, cisplatin. E + C, cisplatin and etoposide in combination.

The H69V and H1184 cell lines were used to assess the possible role of *KEAP1* silencing in SCLC resistance to chemotherapy treatment, using cisplatin and etoposide treatments alone and in combination. The effects of *KEAP1* silencing were evaluated in terms of cellular apoptosis through BCL2 and CAS-3 variations and cell viability. Western blot analysis showed a statistical decrease in silenced *KEAP1* in H69V for both BCL2 and cleaved CAS-3 proteins under etoposide (40 μM, 24 h) or cisplatin (20 μM, 24 h) treatment (Figure 9A,B). A statistically significant increase in cell viability after *KEAP1* silencing under cisplatin (5 μM, 24 h) and etoposide (5 μM, 24 h) treatment was observed in H69V (Figure 9C). A trend in CAS-3 levels was observed in H69V using a combination of the two drugs (etoposide + cisplatin), confirmed by the viability assay (Figure 9C).

By contrast, no significant apoptotic or viability events were observed in treated H1184 cell lines under *KEAP1* silencing (Figure S4).

### 3.6. Effect of KEAP1 Silencing in SCLC Cell Lines under DAPT Treatment

The role of *KEAP1* as a potential predictive marker of response to DAPT treatment was evaluated using the H69V cell line as a model.

Gene expression analysis was performed under DAPT treatment on the H69V *KEAP1*-silenced cell line in the first instance to evaluate the transcript levels of KEAP1, NOTCH1, and its target gene HES1. Western blot analysis was then performed to assess the effect of *KEAP1* silencing on protein levels of KEAP1, NRF2, NOTCH, HES1, E-cadherin, and c-MYC.

**Figure 10:**
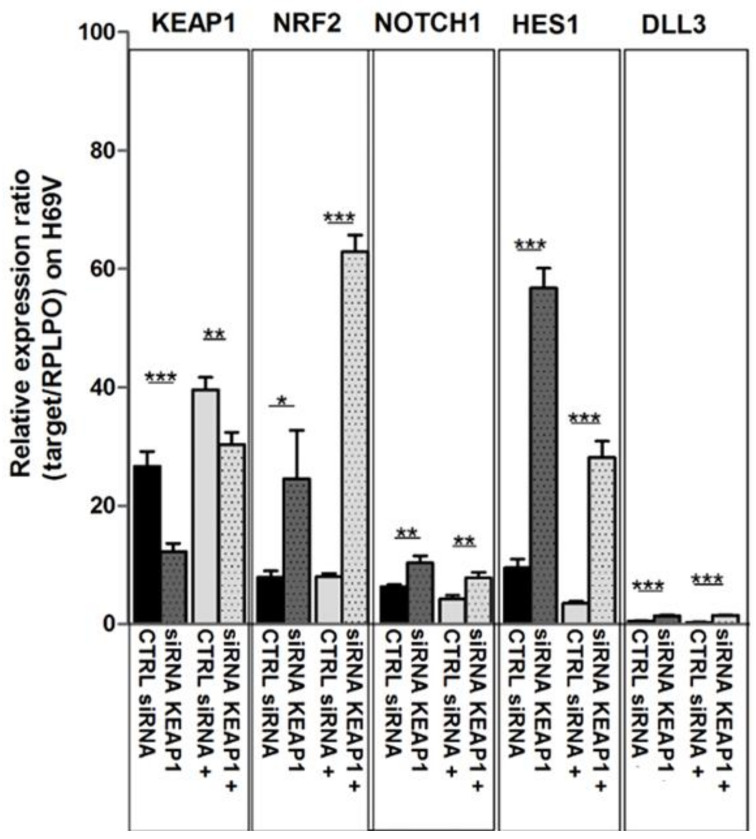
Changes in mRNA transcript levels of KEAP1, NRF2, NOTCH1, and HES1 in H69V cell line after *KEAP1* silencing under DAPT treatment, normalized to *RPLPO*. * *p* < 0.05, ** *p* < 0.01, *** *p* < 0.001 (Student’s *t*-test). siRNA KEAP1+, *KEAP1*-silenced cell under DAPT treatment.

**Figure 11:**
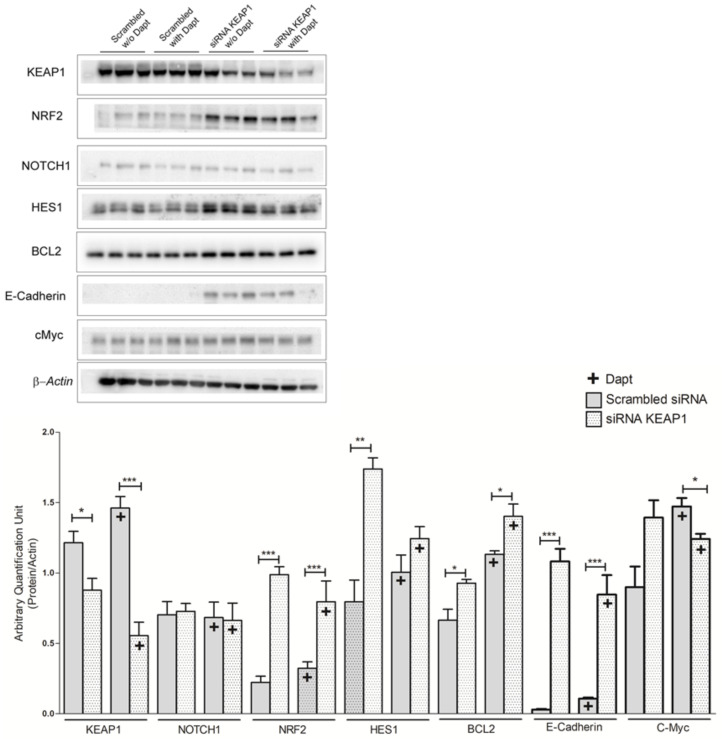
Representative Western blot analysis showing the expression levels of KEAP1, NRF2 NOTCH1, HES1, BCL-2, E-cadherin, and c-MYC proteins in H69V cell line after *KEAP1* silencing under pharmacological DAPT treatment, normalized to actin. * *p* < 0.05, ** *p* < 0.01, *** *p* < 0.001 (Student’s *t*-test). Original western blots are shown in the Supplementary Materials as File S1.

As expected, the results showed that *KEAP1* silencing resulted in a significant decrease of KEAP1 transcript levels followed by a significant increase in the mRNA levels of NRF2. Moreover, *KEAP1* silencing induced a significant increase of NOTCH1, HES1, and DLL3 transcript levels (Figure 10). The same significant effects were also observed at protein level for KEAP1, NRF2, HES-1, E-cadherin, and c-Myc. Significant variation in NOTCH1 protein levels was observed (Figure 11).

No significant variations in viability were noticed after KEAP1 reduction and DAPT treatment at the same time (Figure S5).

## 4. Discussion

NRF2 is a key regulator of the cell adaptive response to radical oxidant species and xenobiotics and exerts its activity through the interaction with its negative regulator, KEAP1. Several studies have suggested that the activation of NRF2 protects against chronic conditions such as cardiovascular diseases, lung inflammation, and nephropathy [33]. However, in recent years, the dark side of NRF2 has emerged and growing evidence suggests that NRF2 constitutive upregulation is associated with cancer development and progression and contributes to both intrinsic and acquired chemo- and radio-resistance [26].

In lung cancer, the deregulation of the NOTCH pathway is mainly ascribable to missense mutations that affect the ligand-binding or ankyrin domains, leading to ligand-independent activation by triggering tumor suppressors [34,35]. In SCLC, the canonical activation of the NOTCH pathway enhances HES1 expression, subsequently suppressing the expression of Achaete-scute homolog 1 (ASCL1) and inhibiting neuroendocrine DLL3-medited differentiation. The expression of ASCL1 determines the molecular subtype classification of SCLC into four groups: SCLC-A (ASCL1-dominant), SCLC-N (NEUROD1-dominant), SCLC-P (POU2F3-dominant), and SCLC-I (SCLC-inflamed). The latter mainly refers to an inflamed state with low expression levels of the previous transcription factors but high expression levels of IFN-γ activation and immune checkpoint molecule expression [36,37], also being correlated to better overall survival (OS) in subgroup analysis of IMpower133 and CASPIAN trials investigating atezolizumab or durvalumab, respectively, plus chemotherapy in SCLC patients with extensive disease stage [7,38].

Here, we investigated the molecular basis of NRF2/NOTCH crosstalk deregulation by *KEAP1* in SCLC and evaluated its impact on impairing responses to conventional chemotherapies and NOTCH inhibitors.

Our presented and previously reported genetic and epigenetic investigations on *KEAP1/NRF2* and *NOTCH* genes in a collection of neuroendocrine cell lines confirmed that *KEAP1* and *NFE2L2* genes mutations only marginally affect the SCLC histology and are typically related to non-small cell lung cancer (NSCLC) [11,30,39]. Only a single recently reported aminoacidic change in the DGR domain of the *KEAP1* gene has in fact been identified in the H1184 cell line and reported to affect the ability of KEAP1 to bind NRF2, promoting its proteasomal degradation in the cytoplasmatic compartment of cells [40]. On the other hand, hypermethylation in the promoter region of *KEAP1* identified in three out of the seven cell lines corroborates the hypothesis of a role of aberrant DNA methylation in the modulation of KEAP1 transcription in SCLC [32].

As an initial result of our in vitro investigations, a correlation between the molecular deregulation of the *KEAP1* gene and the variations in mRNA expression level in SCLC cells was found. Both aberrant DNA methylation and mutation of the *KEAP1* gene in SCLC cell lines were related to cellular localization of KEAP1 and NRF2 proteins and impacted the modulation of KEAP1 at transcript and protein levels. The observed expression of NRF2 target genes in SCLC cell lines was not homogeneous at either transcript or protein levels. This is probably related to both the different genetic backgrounds of the cell lines and to the reported existence of alternative NRF2-independent mechanisms of ARE gene modulation, as for AKR1C1 [41].

Interfering RNA inhibition of the KEAP1 was also conducted to corroborate this first observation and significant variable increases in NRF2 and its target genes AKR1C1, TXN1, and NQO1 at mRNA and protein levels were observed in three different cell lines, as previously reported in other solid tumors [42]. AKR1C1 is a member of the AKR1C family involved in carcinogen metabolism. It is highly expressed in lung tumor tissues and it is characterized by ARE in the promoter region, which is regulated by NRF2 [43]. TXNRD1 is a seleno-protein that plays a role in enzyme catalysis at the active site of the protein and acts on the NRF2 pathway as a component of a redox-sensitive trigger [44]. NQO1, which is tested via gene expression, plays a critical role in quinone metabolism and toxicity and, after dissociation from KEAP1, activated NRF2 enriched in the nucleus and mediated NQO1 induction by binding to endogenous ARE [45]. Globally, these findings corroborate the idea of *NRF2* modulation activity by the KEAP1 protein in an SCLC cell line model.

In H69V and H1184 cell lines, it was clearly observed that *KEAP1* silencing also induced significant increases in NOTCH1, HES1, and DLL3 transcript levels and HES1 protein levels. Although we found elevated levels of HES-1 that were not associated with NOTCH activation, this could be explained by possible mediation via a NOTCH-independent mechanism as well as c-jun N-terminal protein kinase (JNK) signaling, as reported in different previous papers [46,47,48]. It remains to be clarified whether the negative regulatory role of *KEAP1* also significantly modulates the NRF2/NOTCH interplay or whether its activity in SCLC is independent from *KEAP1* activity.

Taking into account the well-known role of the KEAP1/NRF2 pathway in chemotherapy resistance, pharmacological tests were also performed to investigate whether *KEAP1* suppression had an impact on SCLC as a molecular marker to predict tumor cell response to cisplatin and etoposide agents. Results from viability assays and measurements of BCL2 and caspase 3 in the H69V cell line suggest that *KEAP1* silencing effectively affects the drug response, with contrasting effects on tumor cells when the two drugs were used separately. The use of a combination of cisplatin and etoposide appeared to cancel the effect of each. We speculate that the benefit of the pairing seems to come from each drug independently, not from the joint action. While it stands to reason that combining targeted drugs could improve benefits, the rational development of drug combinations against SCLC is still hampered by the limited understanding of underlying cellular processes as a plausible explanation. In this context, additional studies involving monitoring caspase 7 levels and performing proliferation and pharmacological evaluations are demanded to confirm our hypothesis.

Finally, results from the pharmacological treatment of the H69V cell line using DAPT supported the idea that resistance to treatment using NOTCH1 inhibitors could be poorly linked to KEAP1 expression. Under DAPT treatment, *KEAP1*-silenced cells did not in fact show significant variation in NOTCH and HES-1 protein levels or changes in the viability of tumor cells. Scrambled siRNA and *KEAP1* siRNA followed the same trend as those that had been treated with DAPT. It will be necessary to understand whether the increased levels observed were a phenomenon induced by adding DAPT or whether they were caused by other mechanisms dependent on *KEAP1* that contributed indirectly to this upward trend. As reported, an increase (and not a decrease) in scrambled siRNA was observed following DAPT treatment compared with non-treated cells, while cells subjected to *KEAP1* siRNA followed an inversely proportional effect. This could be explained by the fact that DAPT upregulates the expression of E-cadherin. Thus, we could hypothesize that DAPT suppresses EMT-related protein expression and cell proliferation, invasion, and migration, suggesting that it ultimately limits the progression of carcinogenesis [49]. Other interesting results have demonstrated that the inhibition of the NOTCH signaling pathway with DAPT downregulated the expression of Snail and vimentin and upregulated the expression of E-cadherin at the mRNA and protein levels in a time- and concentration-dependent manner [50], which may be consistent with what we found.

In summary, we demonstrated that *KEAP1* silencing induced the upregulation of NRF2 with a consequent increase in SCLC cells’ chemoresistance under cisplatin and etoposide treatment. *KEAP1* modulation also interfered with NOTCH1, HES1, and DLL3 transcription, so we can speculate on the cooperation of these two pathways in tumorigenesis of SCLC.

The limitations of this study are mainly related to the number of cell lines used in our observation and the lack of observations related to *NRF2* silencing. In *KEAP1* mutant cell lines, it has been widely reported, firstly in lung cancer, that KEAP1 hardly regulates NRF2 activation, and NRF2 protein levels are high [51]. In consequence, we can hypothesize that this could be an excellent starting point for investigations in additional cell lines to confirm our speculation that supposes a prognostic significance of KEAP1 in SCLC in clinical settings, and may also be related to NOTCH activity through DLL3 modulation. DLL3 has been found to be highly expressed in SCLC-A and SCLC-N subtypes and it should be of interest to evaluate whether DLL3-targeting therapies achieve better outcomes in this setting [52]. The first clinical experience investigating an ADC targeting DLL3, rovalpituzumab tesirine (Rova-T), was halted after interesting preliminary results in the treatment of SCLC, due to a lack of efficacy in phase 3 studies [16]. Recently, tarlatamab, a bispecific TCE with a dual affinity for DLL3 on tumor cells and CD3 on T cells, showed impressive results in terms of antitumor activity, with durable objective responses and promising survival outcomes in patients with previously treated SCLC [53]. Hence, the predictive role of DLL3 expression for DLL3-targeted treatment continues to be a hotly debated topic with several ongoing trials [16] and finds its roots in a deeper comprehension of SCLC biology [54].

## 5. Conclusions

In the past decade, the efficacy of targeting key “growth drivers” in cancer treatment for a small subset of lung cancers has emerged and encouraged the investigation of new target proteins that are selectively expressed in cancer cells, which should suggest novel, additional pharmacological options.

The key role and molecular impact of KEAP1/NRF2 modulation in SCLC have generally been uninvestigated, especially in the context of NOTCH pathway crosstalk.

Two main points are underlined in this context by our work. Firstly, the molecular background evaluation of dysfunction in the NRF2 and NOTCH pathways represents a first interesting step to verifying and better understanding the features of KEAP1–NRF2/NOTCH interaction and clarifying its role in SCLC tumorigenesis. The hypothesis about the intersection between NRF2 and NOTCH that arises from the experimental evidence might offer an alternative and valuable approach for responding to the unmet clinical need for biological treatments in SCLC patients. Additional evidence relating to different pharmacological treatments on a large set of SCLC cell lines will accelerate this goal.

## Data Availability

The original contributions presented in the study are included in the article/Supplementary Materials.

## Supplementary Materials

The following supporting information can be downloaded at: https://www.mdpi.com/article/10.3390/cancers16101885/s1, Figure S1: Viability assay and IC50 calculation in H69V and H1184 cell lines under etoposide and cisplatin treatment. Figure S2: Representative Western blot analysis showed the expression levels of NOTCH1 protein on H69V cell line under DAPT treatment with increasing concentrations (50 μM, 65 μM 85 μM, 100 μM, 24 h). Figure S3: Representative Western blots analysis showed the expression levels of NOTCH protein in H69V, HH184, N417, Hcc-33, H209 and H1963, respectively. Figure S4: The viability of H1184 cell line after KEAP1 silencing was tested under cisplatin and etoposide treatment. Figure S5: The viability of H69V cell line after KEAP1 silencing and DAPT treatment. File S1: Original Western Blot Images. Table S1: Primers sequence and probes of *KEAP1* and *ACTB* used for QMSP analysis. Table S2: Probes set used for RT-qPCR analysis.
