# Supplementary material for: Effects of KEAP1 Silencing on NRF2 and NOTCH Pathways in SCLC Cell Lines
Source: Cancers (Basel). 2024 May 15;16(10):1885. doi: 10.3390/cancers16101885 (PMC11120002; doi:10.3390/cancers16101885)
Supplement: Supplementary file 1 [file cancers-16-01885-s001.zip › cancers-3002240-supplementary.pdf]

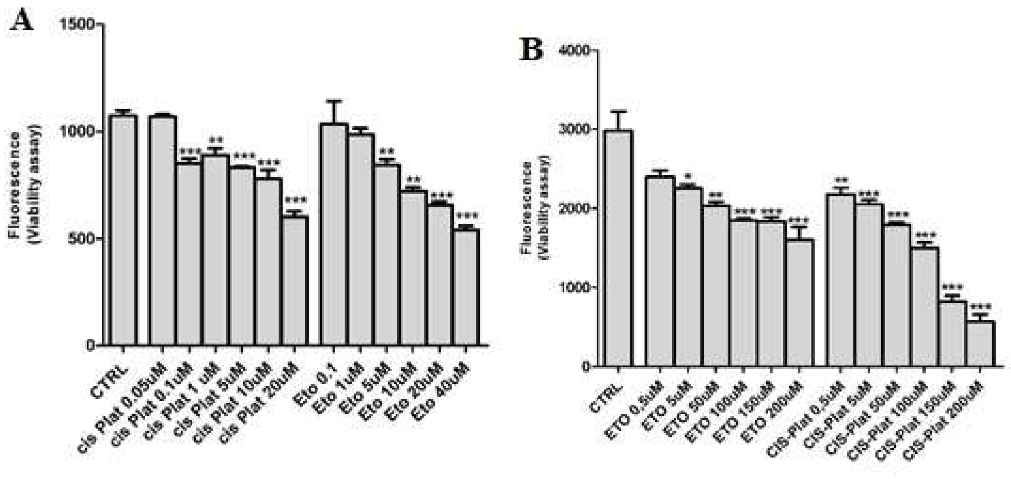

Figure S1. Viability assay and IC50 calculation in H69V and H1184 cell lines under etoposide and cisplatin treatment. To establish the best concentration of drugs to use in the *KEAP1* silencing experiments under drug treatments, the H69V and H1184 cell lines were first treated with increasing concentrations of etoposide and cisplatin. H69V **(A)** and H1184 **(B)** cell lines were treated with six different concentrations of pharmacological compounds for 24h. The data represented means±SE of five treatments. Student's t-test **p<0.05,**p<0.01,***p<0.001*.

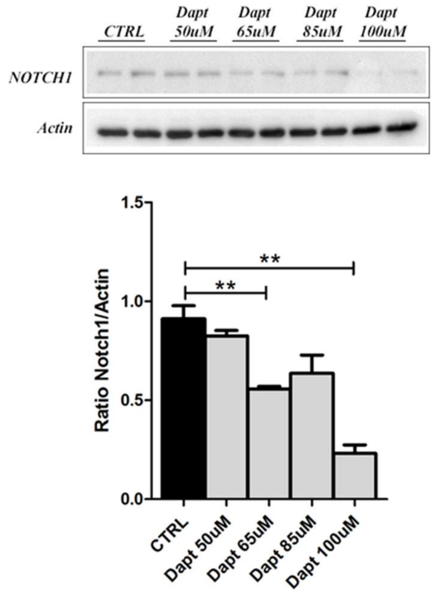

Figure S2. Representative Western blot analysis showed the expression levels of NOTCH1 protein on H69V cell line under DAPT treatment with increasing concentrations (50uM, 65uM 85uM, 100uM, 24h). After 48 hours of transfection, cell line was treated with DAPT and after an additional 24 hours, cell lines were harvested and used to extract RNA and proteins, respectively. After densitometric analysis of the bands which was performed with ImageJ software, the amount of NOTCH1 signaling protein was normalized to actin. **p<0.01 (Student's t-test). siRNA Scrambled (CTRL) and *KEAP1* siRNA.

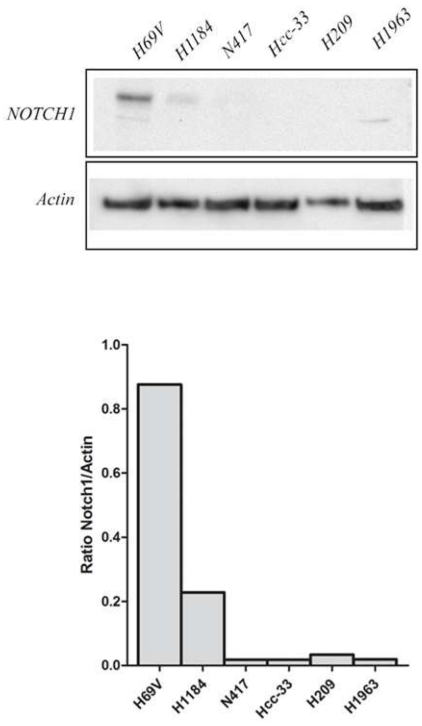

Figure S3. Representative Western blots analysis showed the expression levels of NOTCH protein in H69V, HH184, N417, Hcc-33, H209 and H1963, respectively.

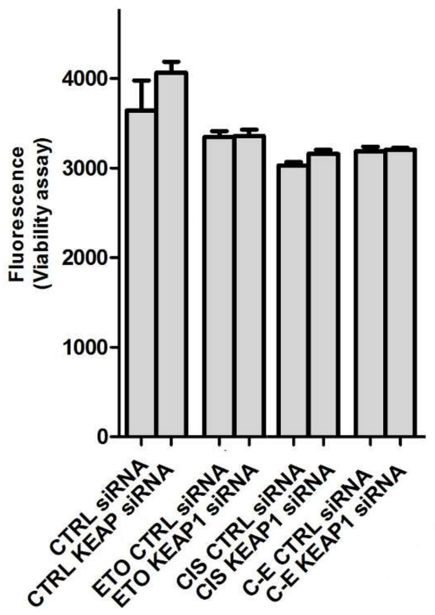

Figure S4. The viability of H1184 cell line after *KEAP1* silencing was tested under cisplatin and etoposide treatment. E, Etoposide. C, Cis-Platin. E+C, Combination.

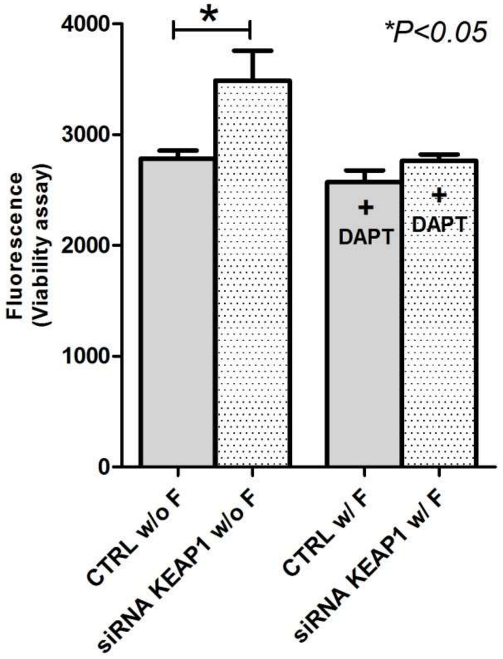

Figure S5. The viability of H69V cell line after *KEAP1* silencing and DAPT treatment. Histograms showed the cells not treated and treated with specific concentrations of pharmacological compound for 36h. * $p<0.05$ (Student's *t*-test).

Table S1. Primers sequence and probes of *KEAP1* and *ACTB* used for QMSP analysis.

Primer/Probe Name	primer sequence (5′ → 3′)	Annealing Temperature (°C)
KEAP1-meth_forward	TGCGGTCGTCGGATTACGAGGTCG	66
KEAP1-meth_reverse	CTTCCATCTCCCGATTTCGTTAC	
KEAP1-meth_probe	FAM-GTGGCGCGTAGTTTCGCGAG-TAMRA	
ACTB-forward	TGGTGATGGAGGAGGTTTAGTAAGT	55
ACTB-reverse	AACCAATAAAACCTACTCCTCCCTTAA	
ACTB-probe	FAM-ACCACCACCCAACACACAATAACAAACACA TAMRA	

Table S2. Probes set used for RT-qPCR analysis.

Gene	TaqMan gene expression
KEAP1	**Hs00202227_m1***
NFE2L2	**Hs00975961_g1***
NOTCH1	**Hs01062014_m1***
HES1	**Hs00172878_m1***
DLL3	**Hs01085096_m1***
NQO1	**Hs02512143_s1***
TXNRD1	**Hs01555214_g1***
RPLPO	**4326314E**

*Taqman gene expression assay from Life Technologies, Thermo Fisher Inc.
