# Supplementary figures and images for: Effects of KEAP1 Silencing on NRF2 and NOTCH Pathways in SCLC Cell Lines
Source: Cancers (Basel). 2024 May 15;16(10):1885. doi: 10.3390/cancers16101885 (PMC11120002; doi:10.3390/cancers16101885)

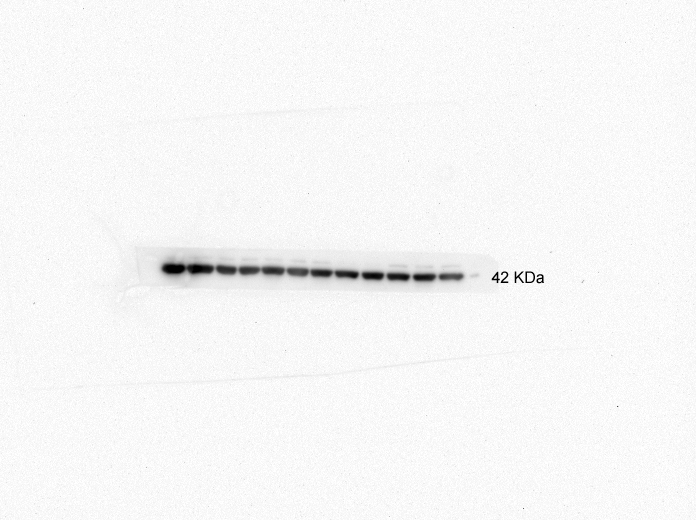

Supplement: Supplementary file 1 [file cancers-16-01885-s001.zip › File S1. Original Western Blot Images/FIG 11/ACTIN/ACTIN.jpg]

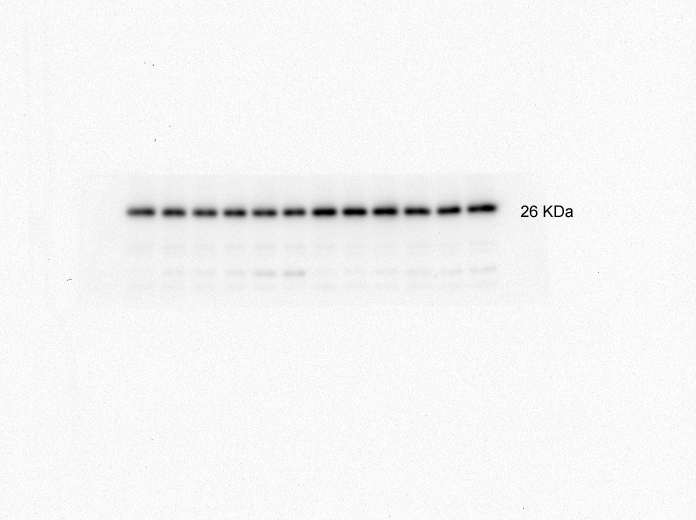

Supplement: Supplementary file 1 [file cancers-16-01885-s001.zip › File S1. Original Western Blot Images/FIG 11/BCL2/BCL2.jpg]

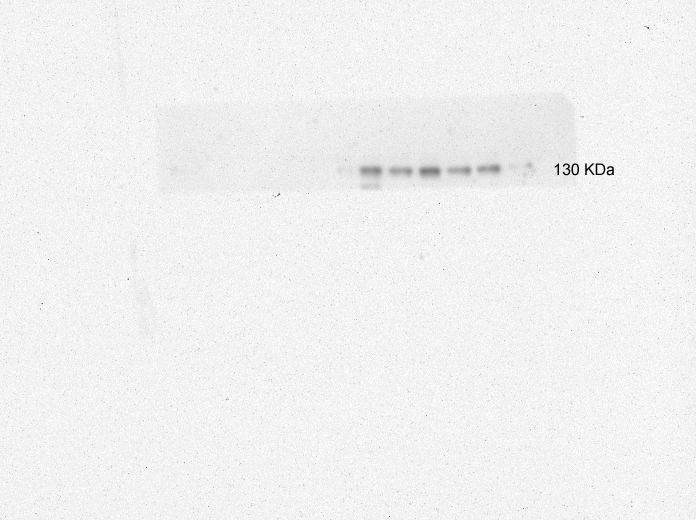

Supplement: Supplementary file 1 [file cancers-16-01885-s001.zip › File S1. Original Western Blot Images/FIG 11/E CAD/e cadherin 2.jpg]

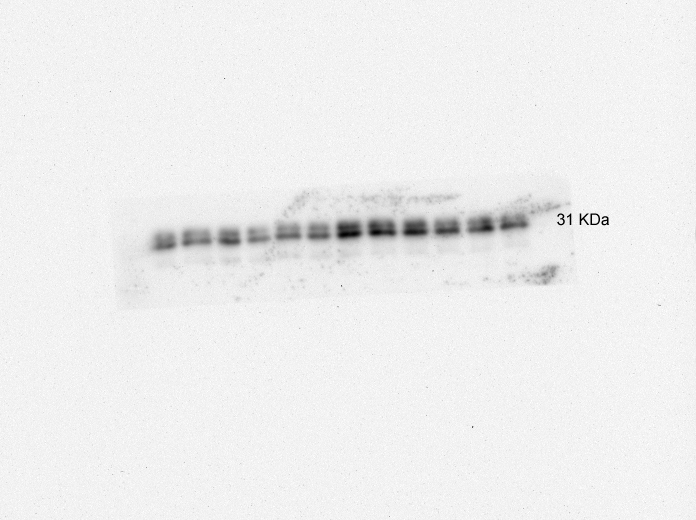

Supplement: Supplementary file 1 [file cancers-16-01885-s001.zip › File S1. Original Western Blot Images/FIG 11/HES1/HES1.jpg]

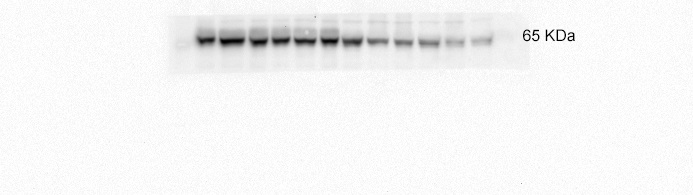

Supplement: Supplementary file 1 [file cancers-16-01885-s001.zip › File S1. Original Western Blot Images/FIG 11/KEAP1/KEAP1.jpg]

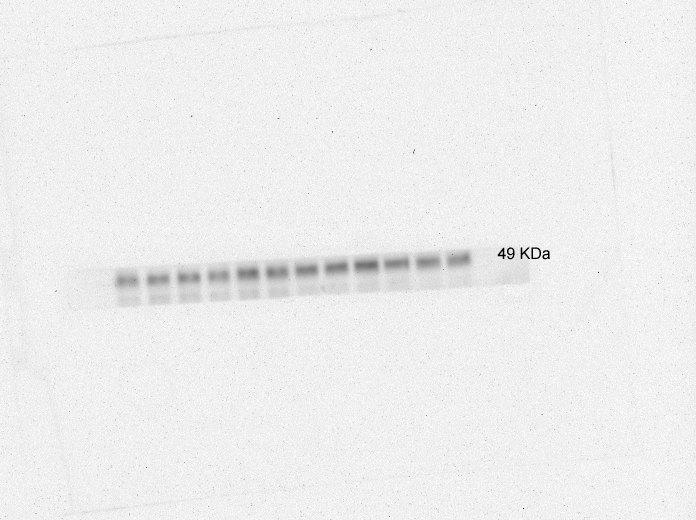

Supplement: Supplementary file 1 [file cancers-16-01885-s001.zip › File S1. Original Western Blot Images/FIG 11/MYC/cMyc.jpg]

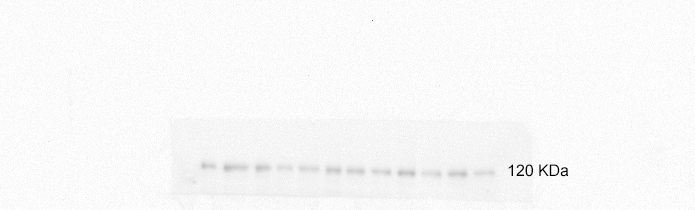

Supplement: Supplementary file 1 [file cancers-16-01885-s001.zip › File S1. Original Western Blot Images/FIG 11/NOTCH1/NOTCH1.jpg]

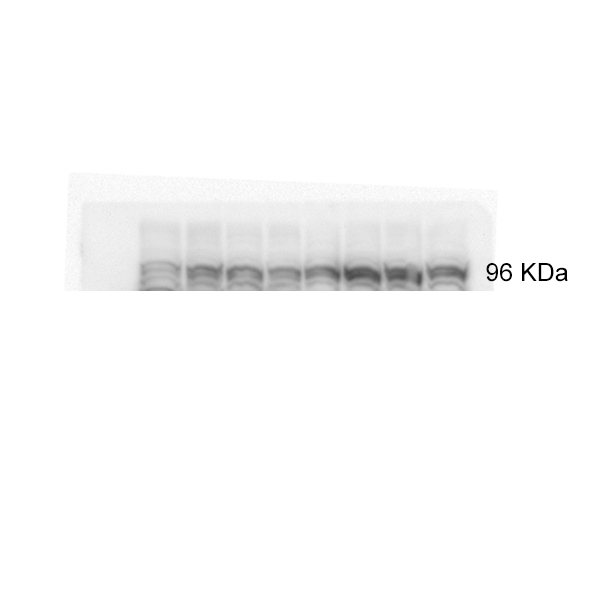

Supplement: Supplementary file 1 [file cancers-16-01885-s001.zip › File S1. Original Western Blot Images/FIG 11/NRF2/NRF2.jpg]

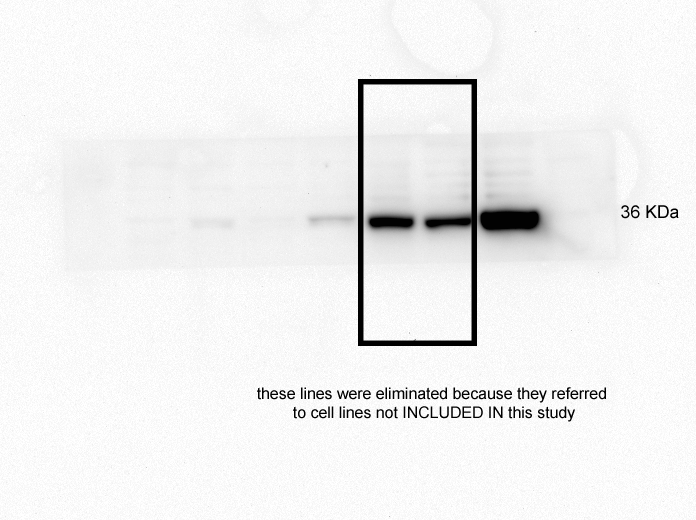

Supplement: Supplementary file 1 [file cancers-16-01885-s001.zip › File S1. Original Western Blot Images/FIG 4/AKR1C1/AKR1C1.tif]

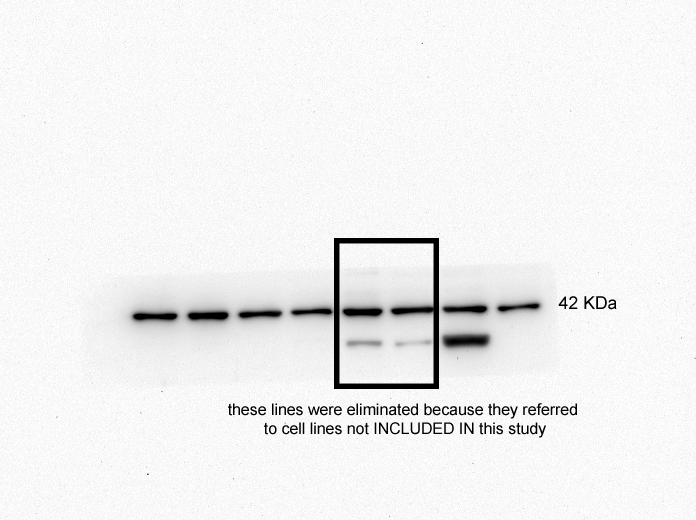

Supplement: Supplementary file 1 [file cancers-16-01885-s001.zip › File S1. Original Western Blot Images/FIG 4/B-Actin/Actin.tif]

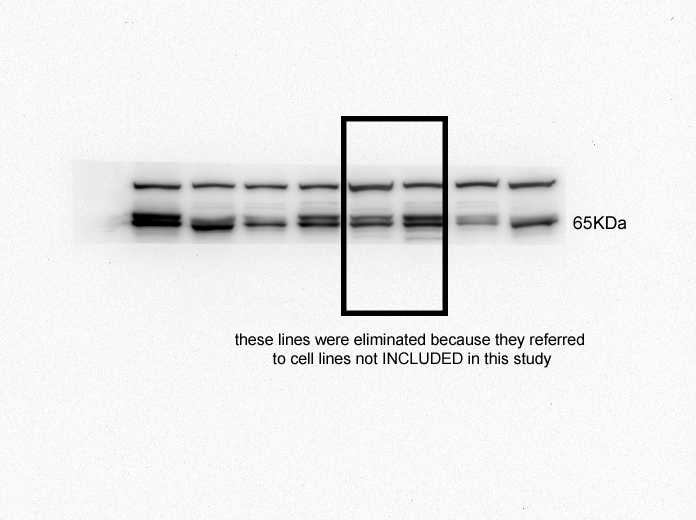

Supplement: Supplementary file 1 [file cancers-16-01885-s001.zip › File S1. Original Western Blot Images/FIG 4/KEAP1/KEAP1.tif]

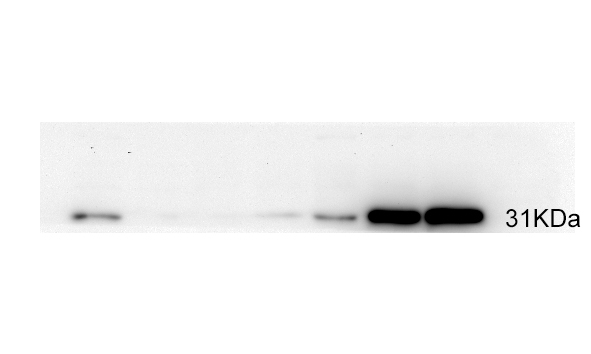

Supplement: Supplementary file 1 [file cancers-16-01885-s001.zip › File S1. Original Western Blot Images/FIG 4/NQO1/NQO1.jpg]

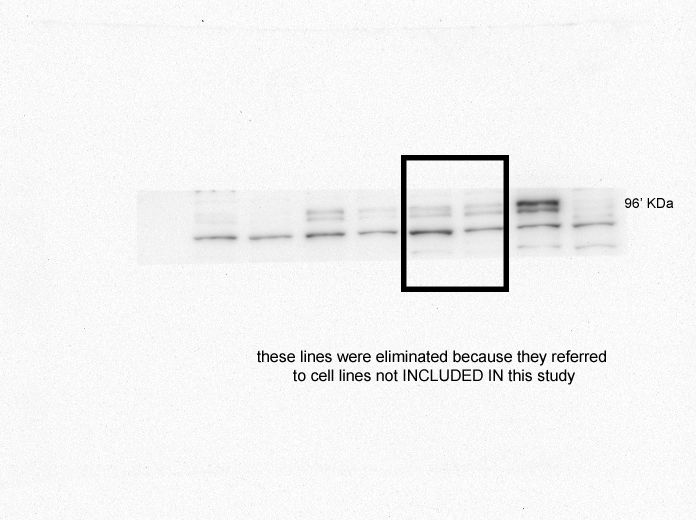

Supplement: Supplementary file 1 [file cancers-16-01885-s001.zip › File S1. Original Western Blot Images/FIG 4/NRF2/NRF2.tif]

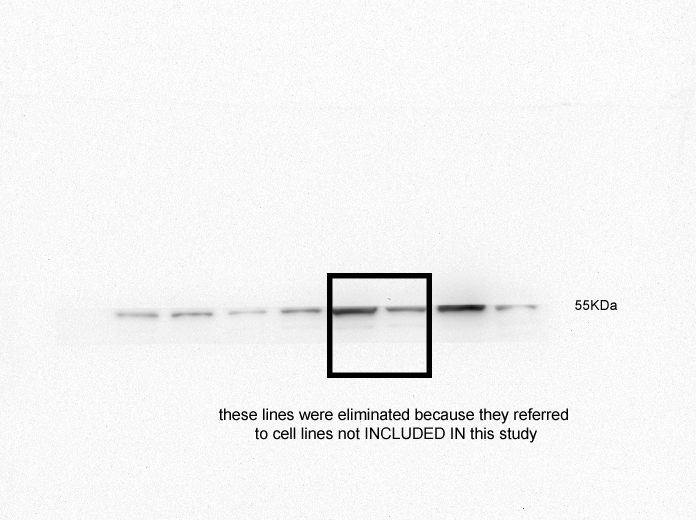

Supplement: Supplementary file 1 [file cancers-16-01885-s001.zip › File S1. Original Western Blot Images/FIG 4/TDXNR1/TXNRD1.tif]

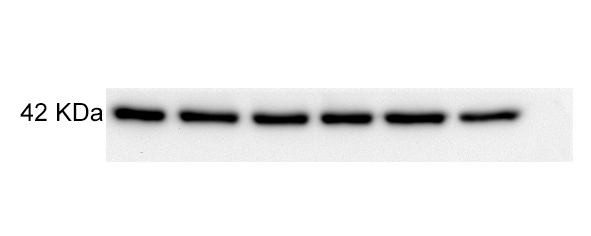

Supplement: Supplementary file 1 [file cancers-16-01885-s001.zip › File S1. Original Western Blot Images/FIG 6 A/H69V/ACTINA/ACTIN.jpg]

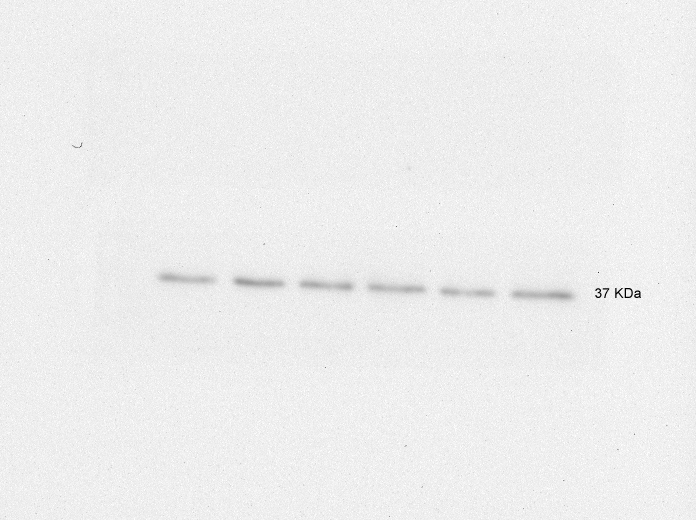

Supplement: Supplementary file 1 [file cancers-16-01885-s001.zip › File S1. Original Western Blot Images/FIG 6 A/H69V/AKR1C1/AKR1C1.jpg]

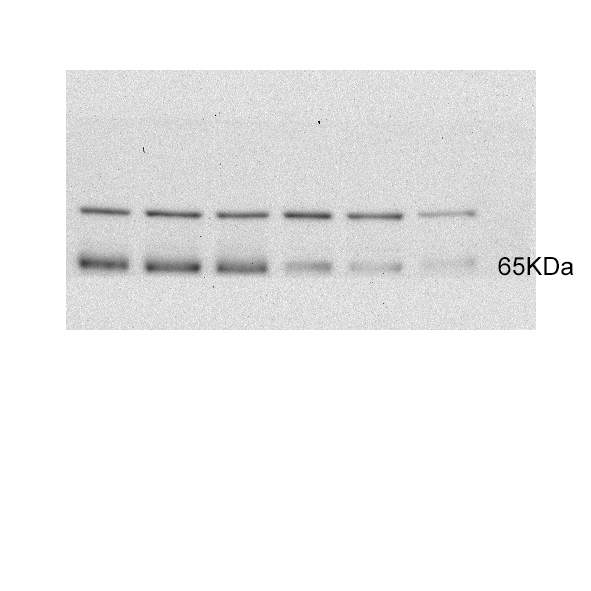

Supplement: Supplementary file 1 [file cancers-16-01885-s001.zip › File S1. Original Western Blot Images/FIG 6 A/H69V/KEAP1/KEAP1.jpg]

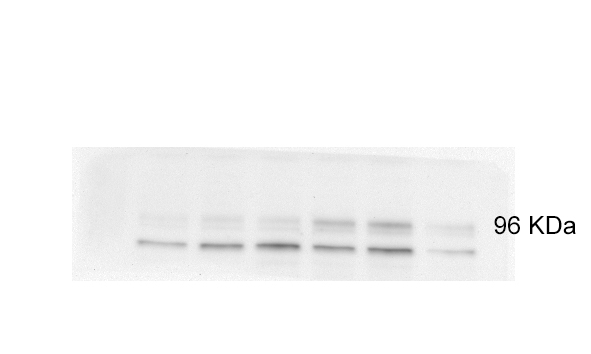

Supplement: Supplementary file 1 [file cancers-16-01885-s001.zip › File S1. Original Western Blot Images/FIG 6 A/H69V/NRF2/nrf2.jpg]

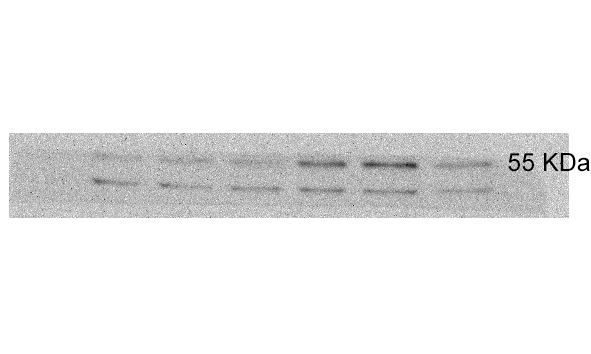

Supplement: Supplementary file 1 [file cancers-16-01885-s001.zip › File S1. Original Western Blot Images/FIG 6 A/H69V/TDXN1/tdxnd1.jpg]

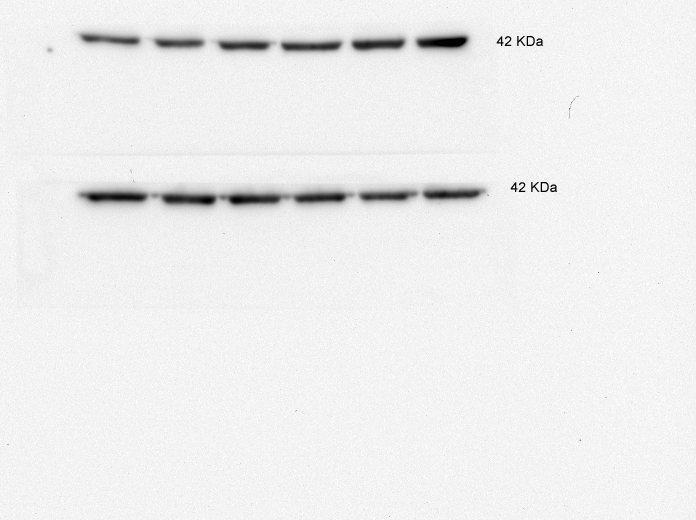

Supplement: Supplementary file 1 [file cancers-16-01885-s001.zip › File S1. Original Western Blot Images/FIG 6 A/N417 -H1184/Actin N417 - H1184/ACTIN N417 - H1184.jpg]

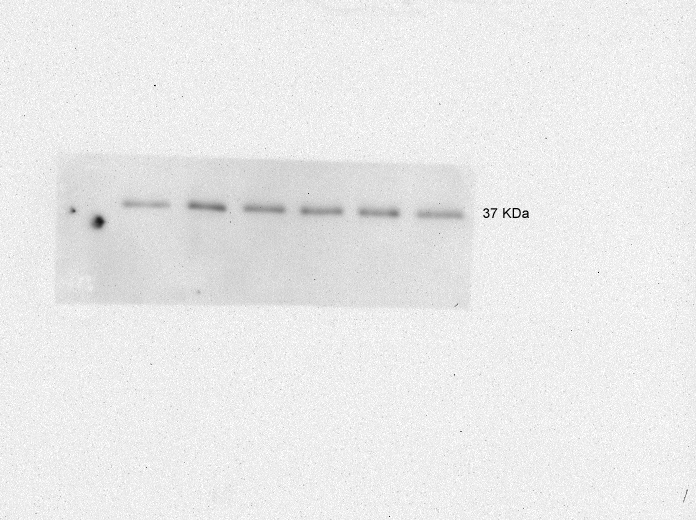

Supplement: Supplementary file 1 [file cancers-16-01885-s001.zip › File S1. Original Western Blot Images/FIG 6 A/N417 -H1184/AKR1C1 N417/AKR1C1.jpg]

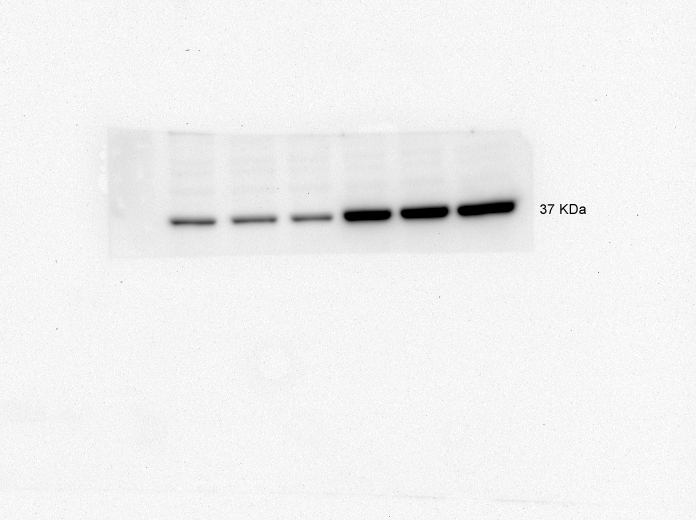

Supplement: Supplementary file 1 [file cancers-16-01885-s001.zip › File S1. Original Western Blot Images/FIG 6 A/N417 -H1184/akr1c1-h1184/AKR1C1.jpg]

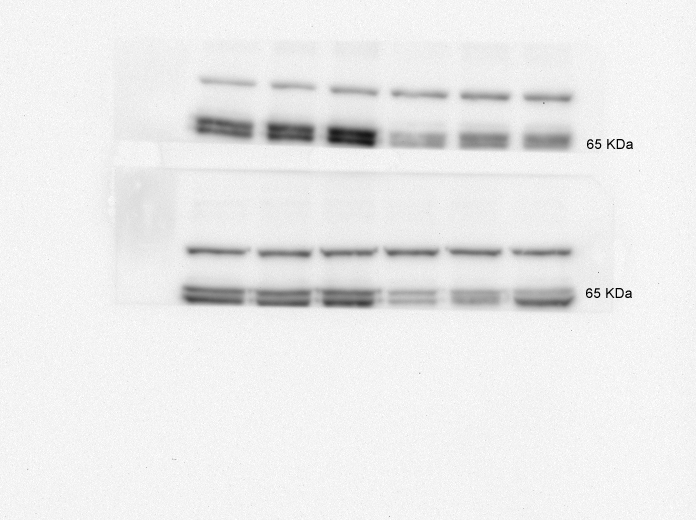

Supplement: Supplementary file 1 [file cancers-16-01885-s001.zip › File S1. Original Western Blot Images/FIG 6 A/N417 -H1184/keap1 N417 - H1184/KEAP1.jpg]

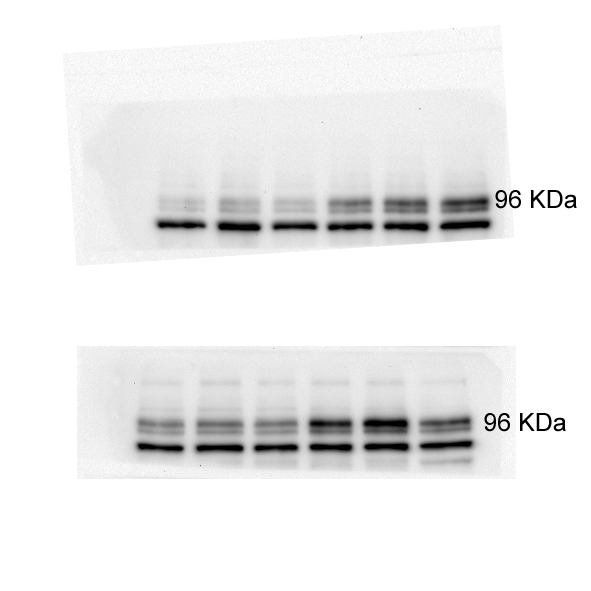

Supplement: Supplementary file 1 [file cancers-16-01885-s001.zip › File S1. Original Western Blot Images/FIG 6 A/N417 -H1184/NRF2 N417 -H1184/NRF2 N417 - H1184.jpg]

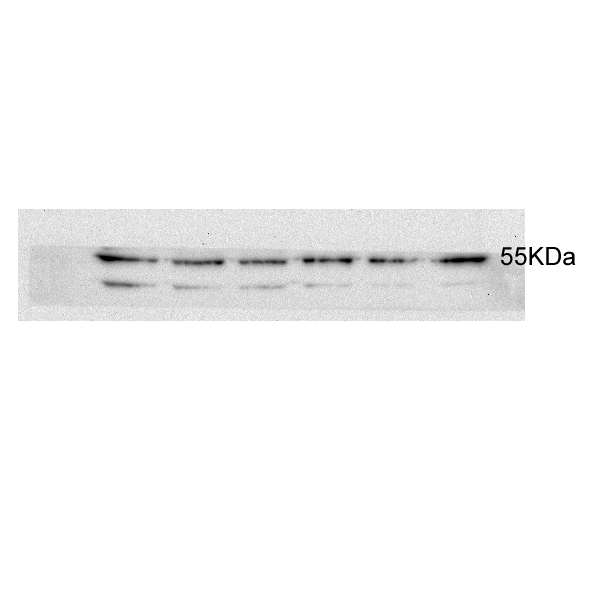

Supplement: Supplementary file 1 [file cancers-16-01885-s001.zip › File S1. Original Western Blot Images/FIG 6 A/N417 -H1184/TDXNRD1 H1184/TDXNRD1 H1184.jpg]

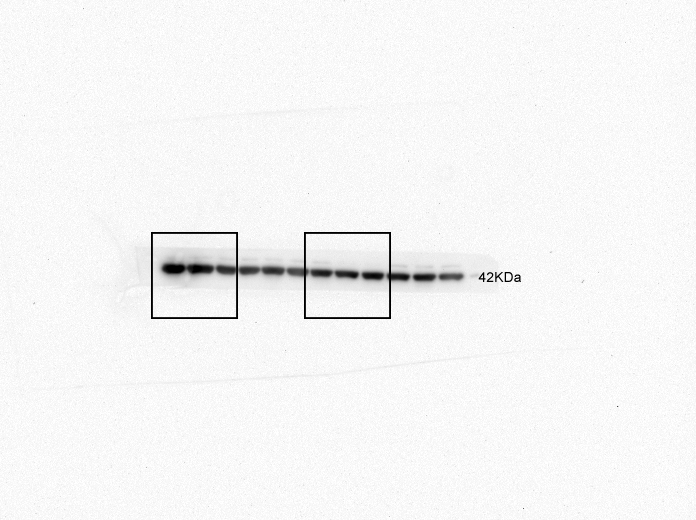

Supplement: Supplementary file 1 [file cancers-16-01885-s001.zip › File S1. Original Western Blot Images/FIG 8 A/H69V/B ACTIN/ACTIN.tif]

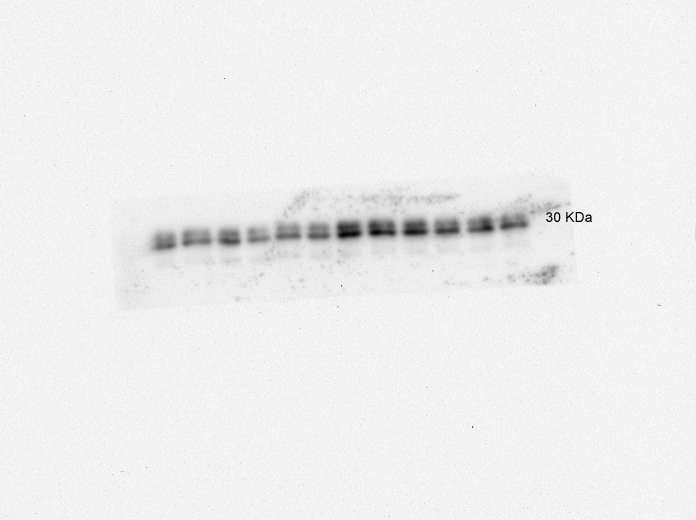

Supplement: Supplementary file 1 [file cancers-16-01885-s001.zip › File S1. Original Western Blot Images/FIG 8 A/H69V/HES1/HES1.tif]

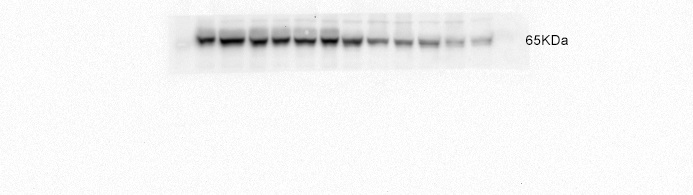

Supplement: Supplementary file 1 [file cancers-16-01885-s001.zip › File S1. Original Western Blot Images/FIG 8 A/H69V/KEAP1/KEAP1/KEAP1.jpg]

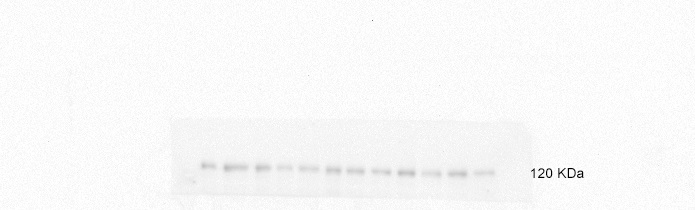

Supplement: Supplementary file 1 [file cancers-16-01885-s001.zip › File S1. Original Western Blot Images/FIG 8 A/H69V/NOTCH1/NOTCH1.jpg]

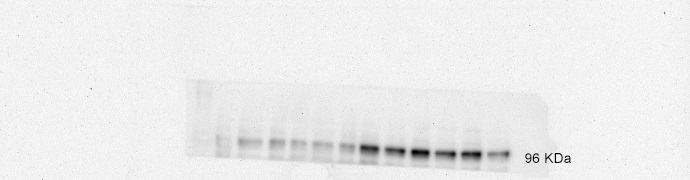

Supplement: Supplementary file 1 [file cancers-16-01885-s001.zip › File S1. Original Western Blot Images/FIG 8 A/H69V/NRF2/NRF2.jpg]

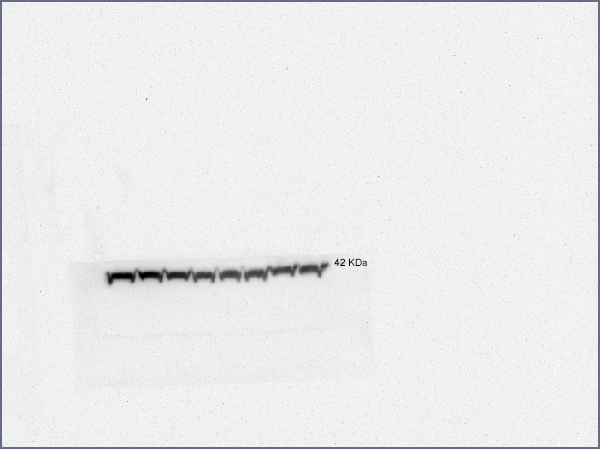

Supplement: Supplementary file 1 [file cancers-16-01885-s001.zip › File S1. Original Western Blot Images/FIG 8 B/H1184/B ACTIN/ACTIN.jpg]

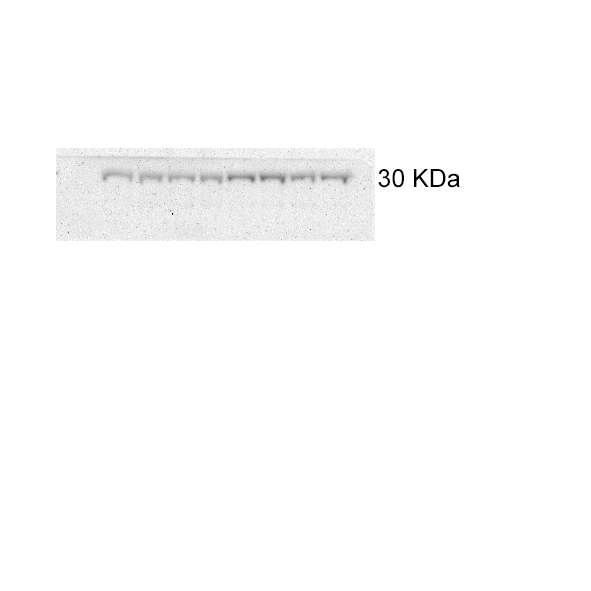

Supplement: Supplementary file 1 [file cancers-16-01885-s001.zip › File S1. Original Western Blot Images/FIG 8 B/H1184/HES1/HES1.jpg]

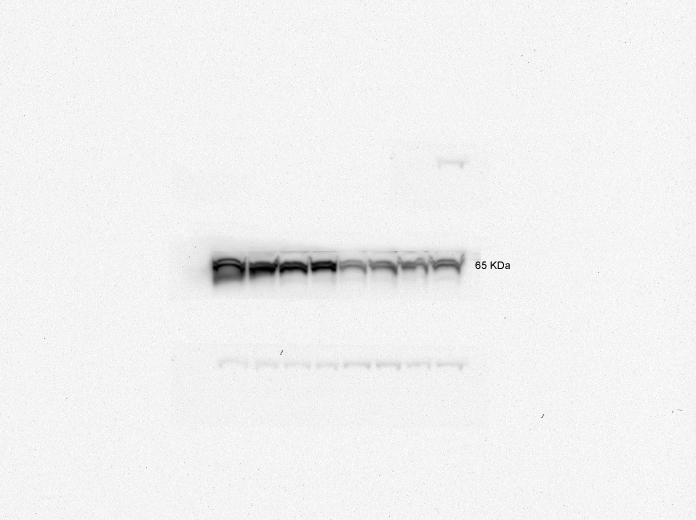

Supplement: Supplementary file 1 [file cancers-16-01885-s001.zip › File S1. Original Western Blot Images/FIG 8 B/H1184/KEAP1/H1184 KEAP1.jpg]

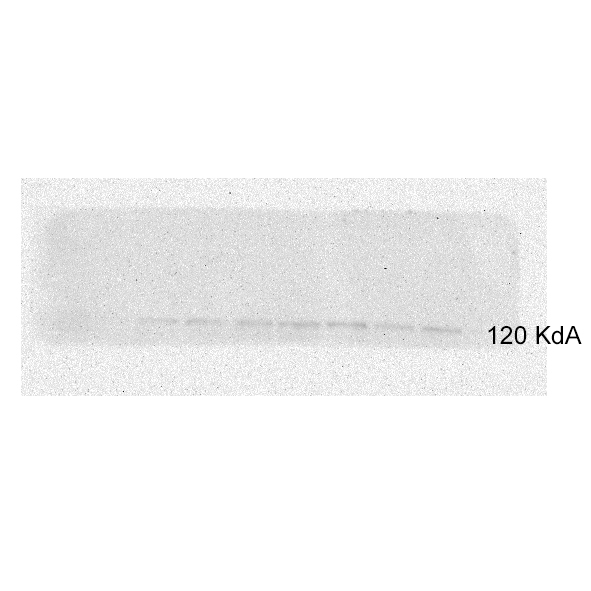

Supplement: Supplementary file 1 [file cancers-16-01885-s001.zip › File S1. Original Western Blot Images/FIG 8 B/H1184/NOTCH1/NOTCH1.jpg]

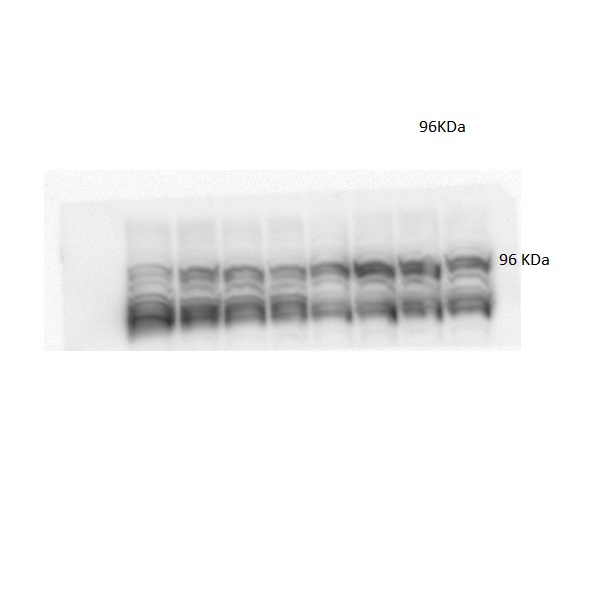

Supplement: Supplementary file 1 [file cancers-16-01885-s001.zip › File S1. Original Western Blot Images/FIG 8 B/H1184/NRF2/NRF2.jpg]

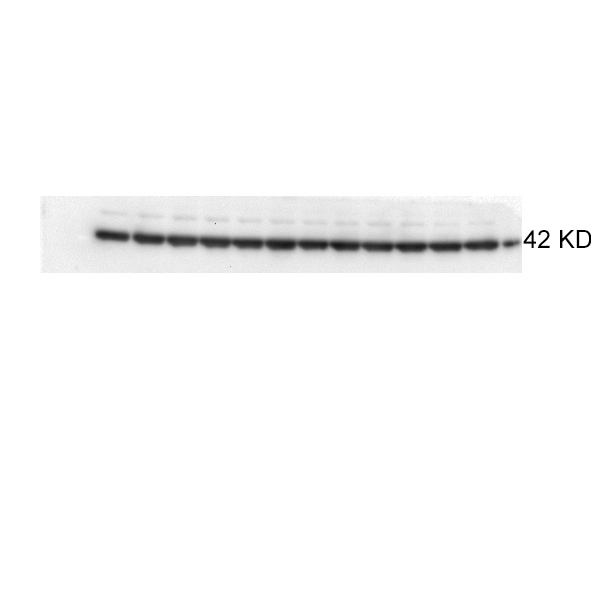

Supplement: Supplementary file 1 [file cancers-16-01885-s001.zip › File S1. Original Western Blot Images/FIG 9 A/ACTIN/ACTIN.jpg]

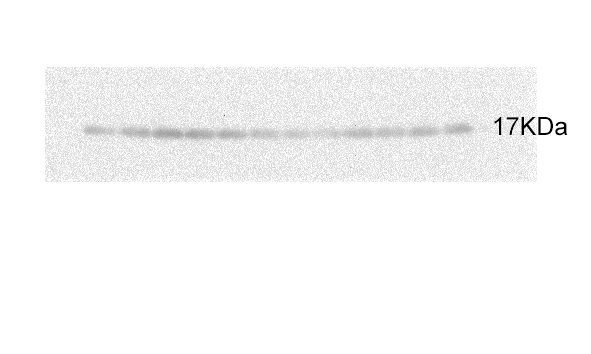

Supplement: Supplementary file 1 [file cancers-16-01885-s001.zip › File S1. Original Western Blot Images/FIG 9 A/CAS 3 CLEAVED/CAS 3 CLEAVED.jpg]

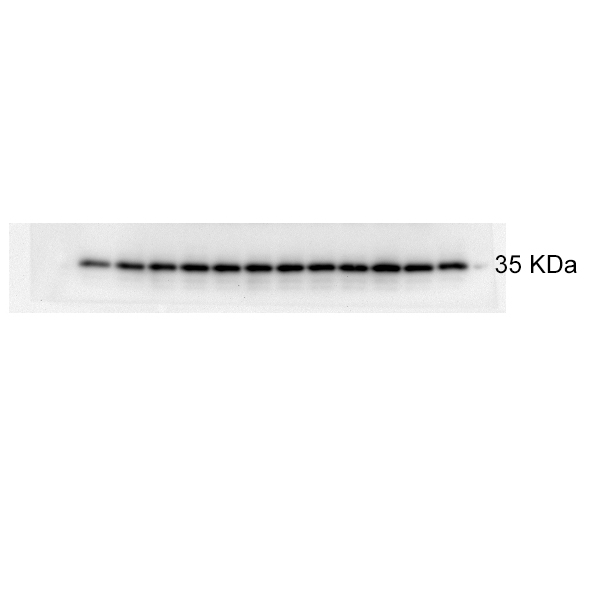

Supplement: Supplementary file 1 [file cancers-16-01885-s001.zip › File S1. Original Western Blot Images/FIG 9 A/CAS 3/CAS3.jpg]

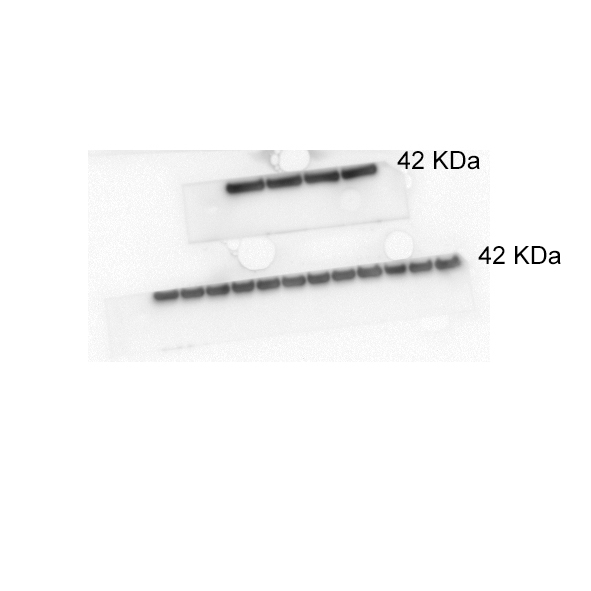

Supplement: Supplementary file 1 [file cancers-16-01885-s001.zip › File S1. Original Western Blot Images/FIG 9 B/ACTIN/ACTIN.jpg]

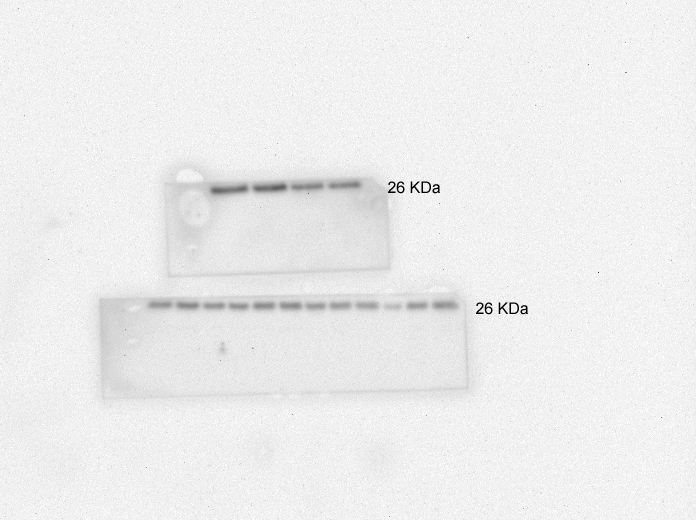

Supplement: Supplementary file 1 [file cancers-16-01885-s001.zip › File S1. Original Western Blot Images/FIG 9 B/BCL2/blc2.jpg]
